# Parentification Vulnerability, Reactivity, Resilience, and Thriving: A Mixed Methods Systematic Literature Review

**DOI:** 10.3390/ijerph20136197

**Published:** 2023-06-21

**Authors:** Jacinda K. Dariotis, Frances R. Chen, Ye Rang Park, Montana K. Nowak, Katherine M. French, Anisa M. Codamon

**Affiliations:** 1 Department of Human Development and Family Studies, College of Agricultural, Consumer and Environmental Sciences, The University of Illinois at Urbana-Champaign, 904 W. Nevada Street, Urbana, IL 61801, USA; yerang@illinois.edu (Y.R.P.); acodam2@illinois.edu (A.M.C.); 2 The Family Resiliency Center, Department of Human Development and Family Studies, College of Agricultural, Consumer and Environmental Sciences, The University of Illinois at Urbana-Champaign, Urbana, IL 61801, USA; 3 Carle Illinois College of Medicine, Beckman Institute for Advanced Science and Technology, Carl R. Woese Institute for Genomic Biology, The University of Illinois at Urbana-Champaign, 506 S Mathews Ave, Urbana, IL 61801, USA; 4 Department of Criminal Justice and Criminology, Georgia State University, Atlanta, GA 30303, USA; rchen6@gsu.edu (F.R.C.); mnowak1@gsu.edu (M.K.N.); kfrench6@student.gsu.edu (K.M.F.); 5 Institute for Interdisciplinary Salivary Bioscience Research, University of California, Irvine, CA 92697, USA

**Keywords:** parentification, role reversal, resilience, coping, systematic review, mixed methods

## Abstract

Parentification occurs when youth are forced to assume developmentally inappropriate parent- or adult-like roles and responsibilities. This review thoroughly examines current empirical research on parentification, its outcomes, and related mechanisms to outline patterns of findings and significant literature gaps. This review is timely in the large context of the COVID-19 pandemic, when pandemic-induced responsibilities and demands on youth, and the shifting family role may exacerbate parentification and its consequences. We used the 2020 updated Preferred Reporting Items for Systematic Review and Meta-Analyses (PRISMA) framework to identify 95 studies (13 qualitative, 81 quantitative, 1 mixed methods) meeting eligibility criteria. Representation from six continents highlights parentification as a global phenomenon. Using thematic analysis, we identified five themes from qualitative studies and five from quantitative studies. These were further integrated into four common themes: (1) some parentified youth experienced positive outcomes (e.g., positive coping), albeit constructs varied; (2) to mitigate additional trauma, youth employed various protective strategies; (3) common negative outcomes experienced by youth included internalizing behaviors, externalizing problems, and compromised physical health; and (4) youths’ characteristics (e.g., rejection sensitivity, attachment style), perceived benefits, and supports influenced parentification outcomes. Future methodological and substantive directions are discussed.

## 1. Introduction

Parentification—also known as adultification, spousification, child carers, or role reversal—occurs when youth are forced to assume developmentally inappropriate parent- or adult-like roles and responsibilities. Definitions highlight that parentification is distinct from supervised or monitored higher-order household responsibilities used by parents to promote positive youth development via leadership skills and character-building. Instead, parentified children and adolescents are expected to become pseudo-parents and pseudo-adults long before they are cognitively and physiologically equipped for these roles. Common roles children assume include household earner, self-carer, family-navigator, language and cultural broker, self-educator, counselor, confidant, caregiver, and emotional supporter (for parents and siblings) [1,2,3].

Floundering, resilient, and thriving outcome trajectories of parentified youth may vary depending on social determinants (e.g., social supports, resources) and perceptions (e.g., fairness, benefits) [4]. The implications of parentification expand beyond the parentified individual (e.g., psychological, cognitive, and physical health outcomes) to the family of origin (e.g., sibling outcomes) and intergenerational transmission [1] if parentified individuals have children (25–40% of parentified women report voluntary childlessness [5]).

The prevalence of parentification in the US is unknown. In 2006, Siskowski and colleagues [6] estimated 1.3–1.4 million parentified 8–18-year-olds (2.9% of the population) in the US, an underestimate according to Hooper and colleagues [3]. More recently, it is reported that 2–8% of youth under age 18 are young carers in high-income countries [7]. A recent study of Polish adolescents reported parentification prevalence estimates exceeding 30% during COVID-19 [8].

Now more than ever, understanding the impacts of parentification is a meaningful undertaking when considering the COVID-19 pandemic. COVID-19 resulted in the sudden loss of multiple resources (e.g., childcare, schooling, employment) due to mandated closures, isolation, quarantine, distancing protocols, and the abrupt loss of family members and friends from illness and death. Since March 2020, when COVID-19 began to rapidly spread within the US, nearly 250,000 children lost a caregiver and households with children were more likely to experience financial hardships including the loss of jobs and health insurance [9]. Education was disrupted for over 1.6 billion children with a projected $17 trillion in life-long earning losses, globally [10]. In short, the COVID-19 pandemic exacerbated contributing factors (e.g., caregiver death; loss of job, income, and health insurance; disrupted education) to parentification.

This systematic review aimed to identify the predictive factors contributing to both positive and negative outcomes of parentification and is timely considering the circumstances forced upon the world by the COVID-19 pandemic. Before delving into the empirical findings, we provide a brief background of the dimensions, sources, and consequences of parentification.

### 1.1. Dimensions of Parentification

Classic models of parentification differentiate types of parentification based on the function it serves, typically as either instrumental or emotional parentification. *Instrumental* parentification involves youth assuming the responsibilities to maintain the household (e.g., meals, chores, finances). *Emotional* parentification requires youth to tend to the emotional needs of family members. This can include becoming a parents’ confidant (e.g., spousification), elevating siblings’ self-esteem, and even promoting harmony among the members. Some parentified youth may fulfill both the instrumental and emotional needs of the family.

Alternatively, researchers have also studied parentification by focusing on the various roles that can be assumed; specifically, parent-focused, sibling-focused, and spouse-focused parentification. The role-based approach emphasizes the role a child takes on, such as becoming a *parent* to care for their own parents (parent-focused) or siblings (sibling-focused), or even a *spouse* to their parents (spouse-focused). The function-based and role-based approaches to parentification are not mutually exclusive concepts, as parent-focused parentification could provide either/or possibly both emotional or instrumental functionality.

### 1.2. Sources of Parentification

Parentification typically results from the intentional or unintentional abdication of parenting responsibilities, child neglect, or child maltreatment by family-of-origin members, especially primary caregivers. The contributing factors of parentification tend to co-occur and there are countless reasons why parents cannot fulfill their role or why children are forced to assume these roles and responsibilities.

Common sources of youth role reversal and role overload include parental illness (e.g., HIV; opioid addiction), parental loss (e.g., death, divorce, incarceration), parental mental illness and physical disability, crises (e.g., displacement via eviction, war, unemployment), dysfunctional family dynamics (e.g., domestic partner violence), and migration (e.g., refugee, immigration). Additionally, parents who were themselves parentified may expect their children to do the same, creating a culture that is passed on for generations [11].

Although the level and degree of impact in the US are unknown, changing US demographics suggest increasing numbers of children are vulnerable to being parentified. In 2019, 26% of youth lived with only one biological parent and 4.0% lived with neither [12]. Parents who work long hours to meet their financial needs may come home with a diminished capacity to attend to household responsibilities and child needs due to fatigue. Living with single parents and parents who experience financial hardships may increase the risk of parentification of these children. With the increasing prevalence of obesity, diabetes, HIV/AIDS, and other chronic health conditions among adults and children, youth in the family without chronic conditions are at greater risk of becoming parentified, tending to the needs of their parents, siblings, or both.

### 1.3. Consequences of Parentification

As articulated in Minuchin’s family system theory [13], a hierarchy of power exists among the family subsystems, and clear hierarchical boundaries between parents and children are considered to be critical to children’s positive development. The dissolution in or the alteration of the family structural boundaries, as may occur when children are parentified and essentially become the parents, breadwinners, and other roles with power in the family, has important implications for children’s behavioral and moral development. Thus, parentification may be linked to adverse behavioral or life outcomes. Parentification is considered particularly harmful when youth are forced to take on tasks beyond their developmental abilities and when they do not receive adequate support [11,14]. Consistent with this proposition, previous research showed that parentified children experience suboptimal outcomes in adulthood, including higher incidence of depression, anxiety, drug use and addiction, under- and un-employment, poor physical health, and lower educational attainment [15,16,17].

In contrast, it has also been argued in early theoretical work that parentification is not necessarily pathological [18]. When youth assume parental roles of moderate intensity in a time-limited manner and their contributions are appreciated, the parentification experience may instead be adaptive [11]. Parentification could beneficially influence youth development because it provides youth with opportunities to master socialization and coping skills, and be self-reliant, which contribute to healthy identity formation and improved self-esteem. In support of this, early parentification among children of parents with HIV/AIDS were found to have better adaptive coping skills 6 years later [19]. Similarly, parentification was linked to less risky sex among high-risk adolescent girls [20] and with resilience in general [17], although the association may be attributable to other factors such as socioeconomic background.

### 1.4. Purpose of Current Review

This review provides a thorough examination of current empirical research on parentification, its positive and negative developmental consequences, the heterogeneity of parentification impacts, and related mechanisms. Through the examination of the empirical research, we then outline significant literature gaps and provide future research directions.

## 2. Materials and Methods

This review was guided by the 2020 updated Preferred Reporting Items for Systematic Review and Meta-Analyses (PRISMA) framework [21,22].

### 2.1. Eligibility Criteria—Inclusion and Exclusion

To be eligible for inclusion, studies had to meet the following criteria: (1) empirical study including quantitative and/or qualitative primary or secondary data analysis; (2) peer-reviewed publication; (3) include at least one developmental or outcome variable (e.g., mental or physical wellbeing, stress coping, leadership skills, substance use, sexual risk-taking, educational attainment, workforce engagement); (4) written or translated into English; and (5) full text available. Studies could include a wide range of designs and types of data including retrospective, prospective, qualitative, quantitative, experimental, quasi-experimental, and non-experimental studies. Studies were excluded if they were (1) theoretical or review articles with no primary or secondary data; (2) not peer-reviewed (book chapters, reports, theses/dissertations, conference proceedings); and (3) exclusively descriptive of parentification types, prevalence, or incidence with no outcomes reported. If a study did not clearly meet inclusion criteria, two or three authors discussed the study until consensus was reached.

For synthesis, studies were grouped in two ways. First, they were stratified by quantitative versus qualitative and mixed. Then, strata were grouped by study focus: outcomes only (positive, negative, both); outcomes and mechanisms (mediators/moderators).

### 2.2. Information Sources

To ensure a comprehensive literature search, three databases were searched on 21 August 2021, for relevant articles: PsycInfo, Academic Search Complete, and Web of Science. American Psychological Association PsycInfo is a database of abstracts and articles related to psychological, social, and behavioral science. Academic Search Complete (EBSCO Publishing) is a scholarly database spanning numerous disciplines and includes both open-access and non-open-access peer-reviewed and grey literature (e.g., books, reports). Web of Science database indexes scholarly products (e.g., articles from over 12,000 journals, 148,000 conference presentations) from physical and social sciences, humanities, and arts.

### 2.3. Search Strategy

For each database, articles available from the inception date until July 2021 were included. MESH and Boolean search terms were used to generate relevant articles. Parentification is known by other related terms, including spousification, adultification, and role reversal. Therefore, these and their related terms (denoted by *) were searched. Further, the search was contingent (using AND) on outcome-related terms (e.g., outcome, resilience, thriving, effect) to answer the research question. Article search results (record data including title, authors, publication date, journal name, and abstract) for resultant articles were exported to Excel and imported into SPSS to remove duplicates. Table 1 summarized search terms by database and the number of resultant articles by the database.

### 2.4. Selection and Data Collection Processes

This dataset was divided across three authors (JKD, FRC, and MKN) for the initial review of titles and abstracts for eligibility. Ambiguous articles were cross-checked by a different reviewer. For a few unclear articles, the three authors (JKD, FRC, and MKN) discussed the article until a consensus was reached about inclusion or exclusion status. Then, the resulting list of articles was divided in half for a full article review and data abstraction (Round 1). Two of the authors (MKN, AMC) independently abstracted descriptive data from articles (participants, study design, data type, parentification measures used, main findings, category of study) and flagged ambiguous articles for review by the first two authors and noted articles that did not meet eligibility criteria. Next, half of the assigned articles were cross-validated by a different reviewer for verification of abstracted data and relevance. Then, the list of articles for each reviewer was stratified by quantitative or qualitative type and randomly divided into Tiers 1, 2, and 3.

The team met to discuss additional data fields to abstract including specific outcomes, positive or negative relationship direction, significance, and future directions. Then, two reviewers read assigned Tier 1 articles, updated existing fields, and abstracted data for new fields (Round 2). These results were reviewed by the first two authors and the full team met to discuss the process of data abstraction. Then, the reviewers (MKN and AMC) continued abstracting data for Tier 2 and Tier 3 articles and flagged any challenging articles for JKD and FRC to review. In Round 3, cited studies discovered through article review that met the inclusion criteria were also included and data were abstracted. Every article was reviewed independently by at least two reviewers.

### 2.5. Data Items

Data items extracted for eligible articles are listed and defined in Table 2. Most data items are standard and are discussed below. Both positive and negative outcomes were included. This scoping review intentionally optimized study inclusion by broadly defining relevant outcomes and not placing temporal restrictions on outcomes (e.g., early childhood, adolescence, adulthood). The exposure variable of interest was parentification and no temporal restrictions were placed on when this experience occurred.

### 2.6. Study Risk of Bias Assessment

Every article was reviewed independently by at least two reviewers. Studies were cross-verified for relevance by at least one additional independent coder. If questions persisted, a third independent coder reviewed the article, and a fourth reviewer was engaged only in ambiguous cases for which their content expertise was needed. As needed, discussions were conducted until a consensus for inclusion was reached.

The Mixed Methods Appraisal Tool (MMAT) [23] facilitated study risk of bias, rigor, and quality assessment. Authors independently assessed each article on five components relevant to the article type (e.g., qualitative, quantitative descriptive) with “yes”, “no”, and “can’t tell” designations. The tool was adapted to include partial credit for some criteria, including screening items that required a specified research question to receive a “yes” designation. If articles included a purpose, aim, or objective statement, they were further reviewed. Further, subcriteria were created for double-barreled or complex criteria. For example, five subcriteria criteria—inclusion, exclusion, response rate mentioned, and sampling strategies detailed—were used to assess whether a study represented the target population for quantitative studies. Partial credit was given, and overall quality ratings were adjusted to no, low, moderate, or high. At least 10% of articles were cross-validated by a second independent reviewer. Interrater questions about meeting criteria and sub-criteria were noted. Discrepancies between reviewers (~5%) were discussed as a team and resolved.

### 2.7. Synthesis Methods

For study characteristics and parentification and outcome measures results, patterns of findings were quantified by count. For substantive findings, we utilized the six steps of thematic analysis outlined by Braun and Clarke [24]. The first three steps—data familiarity, initial coding, theme generation—were undertaken by independent reviewers separately for qualitative studies and quantitative studies. The second three steps—theme review, theme definition and naming, and report development—involved coders across both study types. Additional details for the six steps are as follows. Data familiarity involved reading each study. Independent reviewers initially coded studies using inductive open-coding (identifying major and subcodes and definitions). Themes were generated based on shared or associated codes. Themes were reviewed by the coders and then by the larger team. Once themes were agreed upon, definitions and names were drafted and them finalized. Last, these were written up for the manuscript. Objectivity was promoted via multiple approaches. First, each study was read, coded, and themed by independent reviewers. Summaries of themes were written by reviewers leading each article. Second, themes were generated for qualitative articles and quantitative articles separately followed by integration analyses conducted for the two study types. Last, these themes were reviewed by other team members before team discussions that centered on description meaning and wording, theme consistency and inconsistency by study types, integration of themes across studies, and consensus of final themes to include in the report.

### 2.8. Certainty Assessment

For quantitative studies, MMAT includes criteria related to the use of confounders. For mixed methods studies, one criterion assesses whether study components adhere to quality standards of quantitative and qualitative traditions. For quantitative studies, the criterion was assessed using four sub-criteria: any confounders included, relevant demographic characteristics used, other (non-demographic) confounders (e.g., pre-exposure outcome levels; the age of parentification onset), and use of sophisticated statistics to model potential confounders.

## 3. Results

The result section is organized into several parts. First, the study selection and characteristics are described (Table 3). Second, measurement patterns regarding parentification (Table 4) and outcomes (Table 5) are shown. Third, study findings are presented as themes by design and analytic approaches (quantitative followed by qualitative and mixed). Last, theme patterns are synthesized across the study design.

### 3.1. Included Study Description

#### 3.1.1. Study Selection

**Figure 1:**
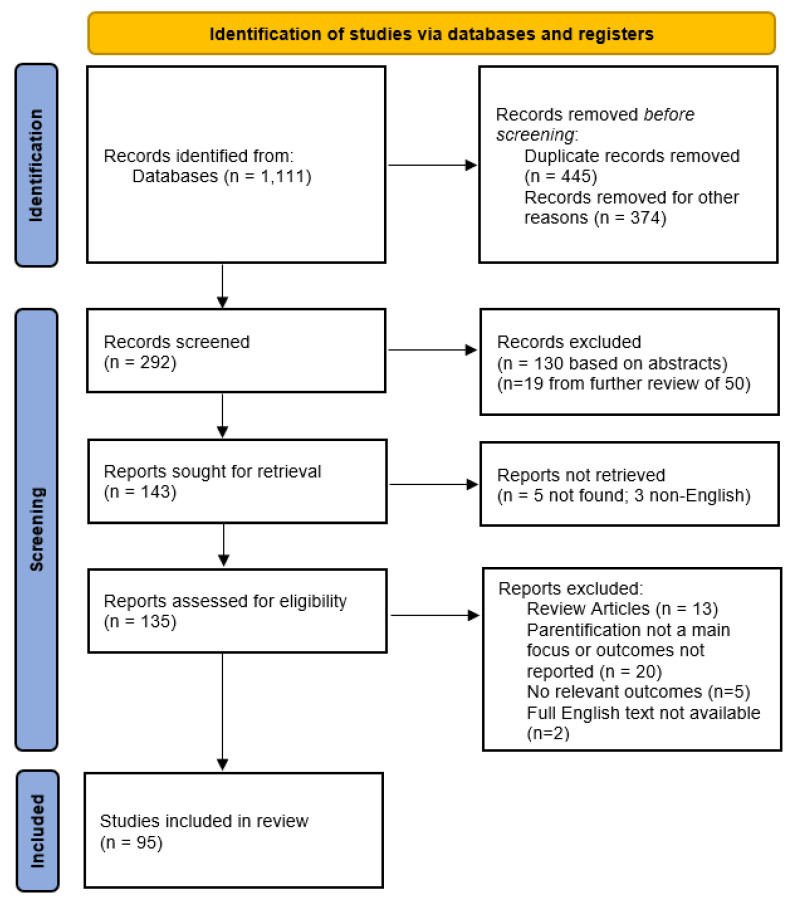
Flow Diagram of Article Search, Screening, Exclusion, and Inclusion.

A total of 1111 articles were retrieved across three databases (227 from PsycInfo; 313 from Academic Search Complete; 571 from Web of Science). Of these, 445 were duplicates and 374 were removed for other reasons (e.g., review articles, no outcomes included, no mention of parentification or related terms or constructs). The manual screening was performed on 292 abstracts and 149 were excluded for not meeting inclusion or meeting exclusion criteria. Of the remaining 143 articles, 135 could be retrieved for eligibility assessment, 40 of which were excluded. The 40 articles were excluded for not meeting the inclusion criteria of our review; specifically, these articles were review articles (*n* = 13), did not include exposure (i.e., at least some participants reporting parentification experiences) or outcome variables (*n* = 21), reported irrelevant outcomes (*n* = 4), or summaries in English but the English full-text article could not be located (*n* = 2). This review focuses on 95 articles successfully screened and meeting all eligibility criteria. The PRISMA flow diagram outlining inclusion and exclusion processes is presented in Figure 1.

#### 3.1.2. Study Characteristics

Table 3 shows study characteristics for one mixed-methods [25], 13 qualitative [26,27,28,29,30,31,32,33,34,35,36,37,38], and 81 quantitative [3,8,17,19,20,39,40,41,42,43,44,45,46,47,48,49,50,51,52,53,54,55,56,57,58,59,60,61,62,63,64,65,66,67,68,69,70,71,72,73,74,75,76,77,78,79,80,81,82,83,84,85,86,87,88,89,90,91,92,93,94,95,96,97,98,99,100,101,102,103,104,105,106,107,108,109,110,111,112,113,114] studies included in this review (*n* = 95; ordered alphabetically within study type). Four papers were published before 2000, 20 between 2000 and 2010, 57 between 2011 and 2019, and 14 after 2020. A total of 55 studies (58%) were conducted in the United States, 19 in Europe (20%), eight in the Middle East (8%), seven in Asia (7%), five in Canada (5%), and one in Africa (1%). All studies used non-random sampling methods to recruit participants.

Studies varied greatly in sample size (quantitative study range: 41–1796; qualitative study range: 2–34; mixed: 128). Among the quantitative (including one mixed-methods) studies, 17 had fewer than 100 participants, 24 had between 100 and 199, 14 had between 200 and 299, and 24 had 300 or more participants. Among the qualitative studies, six had fewer than ten participants and seven had ten or more participants. Forty-five studies included adults, 27 studies included adolescents, and 20 studies included children. Participants’ ages varied across studies, ranging from 19 to 67 among adults, 11 to 21 among adolescents, and three months old to 17 years old among children. Two studies did not explicitly specify the age of the participants. Forty-six studies (48%) sampled school-aged populations (6–18 years old), and 34 studies (35%) focused on college-students, suggesting the use of convenience samples and potential selection effects (e.g., higher income; higher education). Most studies (45%) focused on adult retrospective accounts of parentification experiences. Furthermore, most participants in the studies were female. The overrepresentation of females presents a research gap in understanding parentification among males.

Twenty-six studies included parent-child dyads or triads, 19 of which were mother-child dyads. This disproportionate focus on mother-child dyads reveals a gap within the literature exploring fathers’ and sons’ perspectives on parentification. Six studies (6%) included populations of children with HIV/AIDS-infected mothers. Five studies (5%) included populations of typically developing (TD) siblings who reported having a sibling with autism spectrum disorder (ASD). Five studies (5%) included immigrant and refugee populations. Two studies included clinical populations or cases.

**Table 3:** General Characteristics of Studies.

Author(s)	Year	Sample Size	Participants	Participants’ Age^	Participants’ Sex (Female)	Sample Context	Country	Study Type
Mayseless et al. [25]	2004	128 quan; 16 qual	Adults	37.4 (SD = 12.6)	53.13%	Community sample	Canada	Mixed
Callaghan et al. [26]	2016	2 sibling dyads	Children	range: 7–11	25%	Families affected by domestic violence; case studies drawn from the larger interviews	UK	Qual
Chademana, and van Wyk [27]	2021	7	Children	17.57 (range: 14–20)	14.29%	Selected based on survey findings; orphans living in child- and youth-headed households in Zimbabwe	Zimbabwe	Qual
Chee et al. [28]	2014	5 (mother-child dyads)	Mothers, children	40 (range: 28–54, mothers); 10.4 (range: 7–12, children)	not specified	Low-income families	Singapore	Qual
Collado [29]	2021	10	Young adults	range: 19–23	60%	Convenience sampling; young adult children among internally displaced, refugee families due to political conflict	Philippines	Qual
Gelman and Rhames [30]	2018	4 (mother-child dyads)	Mothers, children	47.5 (range: 43–51, mothers); 18 (range: 15–20, children)	62.50% (children)	Children living at home with a parent with younger-onset Alzheimer’s disease or other dementia	US	Qual
Kabat [31]	1996	2 (mother-daughter dyads)	Adults	18 and 23 (daughters)	100%	Clinical case studies	US	Qual
Keigher et al. [32]	2005	7	Mothers	42 (range: 39–45)	100%	Mothers with HIV	US	Qual
Kosner et al. [33]	2014	34	Adolescents, young adults	16 (range: 15–18, adolescents); 25.70 (range: 23–31, young adults)	64.71%	Young immigrants to Israel from the former Soviet Union during adolescence; adolescents’ current experiences and Young adults’ retrospective accounts	Israel	Qual
Petrowski and Stein [34]	2016	10	Adults	20.1 (SD = 1.37)	100%	College students; women with mothers diagnosed with a long-term mental illness	US	Qual
Rizkalla et al. [35]	2020	23	Mothers	37.62 (SD = 8.93)	100%	Syrian refugee families, mothers’ accounts	US	Qual
Saha [36]	2016	30	Adolescents	range: 11–18	not specified	High school students; middle socioeconomic status; first-born child with siblings	India	Qual
Tahkola et al. [37]	2020	18	Young adults	25.4 (range: 18–32)	77.78%	Finnish young adults with foster care background	Finland	Qual
Tedgård et al. [38]	2019	19	Adults	range: 21–40 (mothers), range: 27–40 (fathers)	68.42%	Parent of children between 1 and 5 years old; parents grew up with drug-abusing parents	Sweden	Qual
Abraham and Stein [39]	2013	116	Adults	19.79 (SD = 2.34)	81%	Emerging adults who have a mother with/without mental illness and poor psychological adjustment	US	Quan
Arellano et al. [40]	2018	1796	Adults	21.23 (SD = 5.25)	79.99%	College students	US	Quan
Baggett et al. [41]	2015	1632	Adults	19.29 (SD = 1.36)	100%	College students	US	Quan
Beffel and Nuttall [42]	2020	108	Adults	20.37 (SD = 1.55)	69.44%	College students; typically developing (TD) who reported having a sibling with Autism Spectrum Disorder (ASD)	US	Quan
Borchet and Lewandowska-Walter [43]	2017	264	Late adolescents	21.39 (SD = 2.52)	87.50%	Individuals	Poland	Quan
Borchet et al. [46]	2016	89 family triads (31 mothers, 27 fathers, 31 late adolescents)	Triads of father, mother and late adolescents	49.58 (SD = 5.14, mothers); 51.04 (SD = 5.32, fathers); 22.58 (SD = 1.52, late adolescents)	53.45% (parents), 64.52% (adolescents)	Family triads; families recruited through students	Poland	Quan
Borchet et al. [8]	2021	191	Adolescents	14.61 (SD = 1.26)	55%	High school students	Poland	Quan
Borchet, Lewandowska-Walter, Polomski, and Peplinska [44]	2020	641	Adolescents	14.96 (SD = 0.36)	60.70%	College students	Poland	Quan
Borchet, Lewandowska-Walter, Polomski, Peplinska, and Hooper [45]	2020	218	Late adolescents	21.37 (SD = 2.49)	86.20%	Majority self-identified college students	Poland	Quan
Boumans & Dorant [47]	2018	297	Adults	18.9 (SD = 1.64)	82.87%	College students; carers/non-carers	Netherlands	Quan
Burton et al. [48]	2018	314	Adolescents	range: 12–13 (63.7%)	50.60%	Middle and high school students	US	Quan
Carroll & Robinson [49]	2000	207	Adults	range: 18–25 (72%)	87.92%	College students; have/do not have alcoholic and/or workaholic parents	US	Quan
Castro et al. [50]	2004	213	Adults	31 (range: 20–59)	85%	College students in clinical and counseling psychology graduate programs	US	Quan
Champion et al. [51]	2009	72 (mother-adolescent dyads; 34 with depression & 38 without depression)	Mothers, adolescents	41.7 (SD = 5.13, mothers); 12.2 (SD = 1.07, adolescents)	50% (adolescents)	Mother with/without history of depression from urban area	US	Quan
Chen and Panebianco [52]	2020	132	Adolescents	14.38 (SD = 2.03)	39.39%	Middle and high school students with at least one parent with a chronic illness	US	Quan
Chen et al. [53]	2018	83	Adults	21.37 (SD = 1.87)	60%	Transitional-aged youth	US	Quan
Cho and Lee [54]	2019	316	Adults	21.86 (range: 18–29)	66.10%	College students	Korea	Quan
Cimsir and Akdogan [55]	2021	147	Adults	20.20 (SD = 1.12)	74.10%	College students	Turkey	Quan
Dragan and Hardt [56]	2016	508 (Poland), 500 (Germany)	Adults	38.7 (SD = 14.4, Poland); 44.8 (SD = 16.1, Germany)	56.3% (Poland), 50.0% (Germany)	Subjects all registered with a market research company	Poland, Germany	Quan
Duval et al. [57]	2018	263	Adolescents	17.08 (SD = 4.45)	78%	High school and college students	Canada	Quan
Fitzgerald et al. [58]	2008	499	Adults	19.29 (SD = 1.95)	100%	College students	US	Quan
Fortin, A., et al. [59]	2011	79 (mother-child dyads)	Mothers, children	37.72 (SD = 5.78, mothers); 10.26 (SD = 1.27, children)	48.10% (children)	Children exposed to domestic violence	Canada	Quan
Godsall et al. [60]	2004	416	Children	14.09 (SD = 1.68)	45.20%	High-functioning/low-functioning children; students	US	Quan
Golan and Goldner [61]	2019	80	Adults	33.47 (SD = 4.76)	100%	Young, first-time Jewish mothers of children aged 12–36 months	Israel	Quan
Goldner et al. [62]	2017	351	Adolescents	14.00 (SD = 0.69)	53%	Middle school students	Israel	Quan
Goldner et al. [63]	2019	334	Adolescents	13.95 (SD = 0.69)	55%	Convenience sample; drawn from mid- to high-SES middle schools	Israel	Quan
Hoffman and Shrira [64]	2019	341 (parent-adult dyads)	Adults	80.05 (SD = 6.10, parents); 53.50 (SD = 5.57, children)	65.4% (parents), 64.2% (adult offspring)	Community sample; Jewish parents of European origin born before 1945 and their offspring born after 1945; parents were alive during World War II and either Holocaust survivors or had no Holocaust background	Israel	Quan
Hooper, Doehler et al. [65]	2012	51 (parent-adolescent dyads)	Parents, adolescents	41.74 (SD = 6.64, parents); 13.80 (SD = 1.28, adolescent)	92% (parents), 51% (children)	Rural community sample	US	Quan
Hooper et al. [17]	2008	156	Adults	22.45 (SD = 6.04)	69.20%	College students	US	Quan
Hooper et al. [66]	2015	977	Adults	21.39 (SD = 5.84)	81%	College students	US	Quan
Hooper, Wallace et al. [3]	2012	314	Adults	22.57 (SD = 6.19, Black); 20.37 (SD = 1.91, White)	56.05%	College students	US	Quan
Jankowski and Hooper [67]	2014	565	Adults	20.78 (SD = 3.79)	81.20%	College students	US	Quan
Jankowski et al. [68]	2013	783	Adults	20.92 (SD = 3.73)	76.40%	College students	US	Quan
Katz et al. [69]	2009	163	Adults	>18 (not specified)	100%	College students; grew up in an intact family with one mother and one father	US	Quan
Khafi et al. [70]	2014	143 (mother-child dyads)	Mothers, children	10.17 (SD = 1.59, children, T1); 14.89 (SD = 1.60, children, T2)	52.40% (children)	Sample overrepresents mothers with anxiety, affective, and/or substance use disorders; predominantly low-income	US	Quan
King and Mallinckrodt [71]	2000	65	Adults	22.41 (SD = 3.21, clinical); 21.53 (SD = 1.64, nonclinical)	85% (clinical), 69% (nonclinical)	College students; clinical/nonclinical samples	US	Quan
Lester et al. [72]	2010	264 (mother- adolescent dyads)	Mothers, adolescent	40.6 (SD = 5.78, mothers); 15.6 (SD = 2.4, adolescent)	58% (adolescent)	Adolescent with HIV/AIDS-infected mothers, or adolescent of neighborhood control mothers	US	Quan
Macfie et al. [74]	2008	138 families	Families (mothers, fathers, children)	27 (range: 18–35, mothers); prenatally, 3 mos., 12 mos., 24 mos., 60 mos., 70 mos. (children)	54.35% (children)	First-time parents	US	Quan
Macfie et al. [73]	2005	57	Families (mothers, fathers, adolescents)	27 mos. (children, Wave 1); 70 mos. (children, Wave 2); 26 (mothers); 28 (fathers)	52.63% (children)	Rural families; waves of data collected on families at 27 months and 70 months from the child’s birth.	US	Quan
Madden and Shaffer [75]	2016	52	Adults	19.49 (SD = 1.39)	80.70%	College students	US	Quan
McGauran et al. [76]	2019	137	Adults	36.90 (SD = 13.91, offender from probation service); 31.83 (SD = 13.25, non-offender)	8% (offender), 69% (non-offender)	Offender/non-offender samples; all white	UK	Quan
McMahon and Luthar [77]	2007	356 (mother-child dyads)	Mothers, children	38.23 (SD = 6.20, mothers); 12.09 (SD = 2.80, children)	54% (children)	Urban, low-income children living with biological mothers; includes mothers (a) with drug problem, (b) with psychiatric problem, or (c) none of the two	US	Quan
Murrin et al. [78]	2021	108	Adults	20.37 (SD = 1.55)	69.44%	College students; typically developing (TD) who reported having a sibling with Autism Spectrum Disorder (ASD)	US	Quan
Nebbitt and Lombe [79]	2010	238	Adolescents	15.62 (SD = 2.08)	47.48%	African Americans living in urban, public housing developments	US	Quan
Nuttall et al. [82]	2012	374 (mother-child dyads)	Mothers, children	21.47 (SD = 5.32, mothers); prenatally, 12 mos., 36 mos. (children)	49% (children)	Community sample; high-risk, first-time adolescent and adult mothers	US	Quan
Nuttall, Ballinger et al. [80]	2021	374 (mother-child dyads)	Mothers, children	21.47 (SD = 5.32, mothers); 36 mos. (children)	49% (children)	Majority of mother sample were non-White (78.4%) and unmarried (74%)	US	Quan
Nuttall et al. [81]	2018	108	Adults	20.37 (SD = 1.55)	69.44%	College students; typically developing (TD) who reported having a sibling with Autism Spectrum Disorder (ASD)	US	Quan
Nuttall et al. [84]	2019	110 mother-child dyads	Mothers, children	30.76 (SD = 7.04)	55%	Predominantly low-income and ethnic minorities; college students; psychosis-proneness sample	US	Quan
Nuttall, Valentino, et al. [83]	2021	235 family triads	Families (mothers, fathers, children)	35.02 (SD = 5.60, mothers, Wave 1); 36.84 (SD = 6.15, fathers, Wave 1); 6.00 (SD = 0.48, children, Wave 1)	45% (children)	Data collected when children were in kindergarten (Wave 1), first grade (Wave 2), and second grade (Wave 3)	US	Quan
Oznobishin and Kurman [85]	2009	184 (Study 1), 180 (Study 2)	Adults, adolescents	23.73 (SD = 2.23, Study 1); 16.73 (SD = 0.94, Study 2)	60.87% (Study 1), 57.78% (Study 2)	College students (Study 1) and high school students (Study 2); both studies include immigrant or Israeli-born	Israel	Quan
Peris et al. [86]	2008	83 family triads	Families (mothers, fathers, adolescents)	15.26 (range: 14–18, adolescents)	48% (children)	Family triads, longitudinal design	US	Quan
Perrin et al. [87]	2013	120	Adults	19.4 (SD = 1.52)	57.67%	College students	Canada	Quan
Prussien et al. [88]	2018	78 (mother-child dyads)	Mothers, children	38.68 (SD = 7.52, mothers); 10.35 (SD = 3.67, children)	45% (children)	Mothers with children diagnosed with cancer	US	Quan
Rodriguez and Margolin [89]	2018	80 (mother-adolescent dyads)	Mothers, adolescents	16 (SD = 1.2)	53.75% (children)	Adolescents in active-duty military families	US	Quan
Rogers and Lowrie [90]	2016	226	Adults	39.0 (SD = 16.3)	50.90%	Age ranged from 19 to 92 years old; 82.4% Caucasian	UK	Quan
Sang et al. [20]	2014	176 (mother-daughter dyads)	Mothers, daughters	40.89 (SD = 7.13, mothers); 15.8 (SD = 1.55, daughters)	100%	African American and Hispanic mother; HIV-negative daughter; low-income inner-city, recruited in agencies that provided services to HIV-infected women; victims of intimate partner violence, and those in substance use recovery	US	Quan
Schier et al. [91]	2015	500 (extraction), 500 (cross-validation)	Adults	44.8 (SD = 16.1; extraction), 39.3 (SD = 11.2; cross-validation)	50% (extraction), 55% (cross-validation)	Internet survey; extraction and cross-validation samples	Germany	Quan
Shaffer and Egeland [92]	2011	196 (mother-offspring dyads)	Mothers, offsprings	Longitudinal: offspring followed from 24 mos initially to adolescent years (age 13, 16, 17.5 years)	42.85% (offspring)	Mother of low socioeconomic status recruited through a public health clinic for prenatal care	US	Quan
Sheinbaum et al. [93]	2015	214	Adults	21.4 (SD = 2.4)	78%	College students	Spain	Quan
Shin and Hecht [94]	2012	697, 605, and 526 across Waves 4, 5, 6	Adolescents	12.31 (SD = 0.58)	53%	Mexican-heritage; middle school students; use Wave 4–6 only	US	Quan
Stein et al. [95]	1999	183 (parent-adolescent dyads)	Parents, adolescents	37.67 (SD = 5.64, parents); 14.75 (SD = 2.07, children)	80% (parents), 54% (adolescent)	Non-infected adolescents of parents with AIDS	US	Quan
Stein et al. [19]	2007	213	Adolescents	14.9 (range: 11–19)	56%	Children with HIV/AIDS-infected mothers	US	Quan
Sullivan et al. [96]	2018	1441	Adolescents	grades 7, 9, and 11 (proxy for age)	50.87%	Middle and high school students	US	Quan
Telzer and Fuligni [97]	2009	752	Adolescents	14.88 (SD = 0.39)	not provided	High school students; ethnically diverse sample of adolescents from predominantly Latin American, Asian, and European backgrounds	US	Quan
Titzmann [98]	2012	382 (mother-adolescent dyads)	Mothers, adolescent	15.2 (SD = 2.55, adolescent)	56.54% (children)	Ethnic (185) and 197 native German families	Germany	Quan
Titzmann and Gniewosz [99]	2018	185 (mother-child dyads)	Mothers, children	15.7 (SD = 2.7, children)	60.4% (children)	Ethnic German immigrant mother-adolescent dyads from the former Soviet Union	Germany	Quan
Tomeny et al. [101]	2017a	60	Adults	29.65 (SD = 13.17)	85%	College students; typically developing (TD) who reported having a sibling with Autism Spectrum Disorder (ASD)	US	Quan
Tomeny et al. [100]	2017b	41	Adults	25.83 (SD = 5.36)	80%	Typically developing (TD) who reported having a sibling with Autism Spectrum Disorder (ASD)	US	Quan
Tompkins [102]	2007	43 (mother-child dyads)	Mothers, children	12.8 (range: 9–16, children)	not specified	Children with HIV/AIDS-infected mother (23) vs. children with HIV-seronegative mother (20).	US	Quan
van der Mijl and Vingerhoet [103]	2017	265	Adults	20.2 (SD = 3.1)	73%	College students	Netherlands	Quan
Van Loon et al. [104]	2017	118	Adolescents	13.47 (SD = 1.40)	50.80%	Adolescents living with a parent with mental health problems	Netherlands	Quan
Walsh et al. [105]	2006	140 (Study 1), 123 (Study 2)	Adolescents	16.8 (SD = 5.60, Study 1); 16.96 (SD = 1.39, Study 2)	45.7% (Study 1), 47% (Study 2)	Study 1: Immigrants from former Soviet Union in Israel vs. Israel born; Study 2: Immigrants from former Soviet Union in Israel	Israel	Quan
Wang et al. [106]	2017	1073	Children	range: 9–17.7 (grades 3–12)	51.80%	Two elementary school and three high school students	China	Quan
Wei et al. [114]	2020	1648	Adolescents	Junior and senior high students (age not specified)	46.30%	Junior and senior high school students	Taiwan	Quan
Wells and Jones [108]	1998	124	Adults	21 (range: 17–48)	65%	College students	US	Quan
Wells and Jones [109]	2000	197	Adults	21 (range: 17–38)	65%	College students	US	Quan
Wells et al. [107]	1999	200	Adults	21 (range: 17–48)	65%	College students	US	Quan
Williams and Francis [110]	2010	99	Adults	23.76 (SD = 5.55)	84%	College students	Canada	Quan
Woolgar and Murray [111]	2010	94 (55 depressed and 39 nondepressed mother-child dyads)	Mothers, children	60.3 mos. (SD = 0.84, index); 60.5 mos. (SD = 0.94, control)	46% (index children), 53% (control children)	Community sample; children and mothers with/without postnatal depression; index and control groups	UK	Quan
Yew et al. [112]	2017	419	Adults	21.9 (SD = 2.04)	62.80%	College students in clinical and nonclinical academic programs	Malaysia	Quan
Zvara et al. [113]	2018	557 (mother-child dyads)	Mothers, children	25.6 (SD = 6.1, mothers); 7.7 (SD = 1.5, children)	49.6% (children)	Rural, low-income families	US	Quan

^ For participant age, range is reported if mean and standard deviation are not provided. Notes: Qual = qualitative; Quan = quantitative; Mixed = mixed methods. Lester, P., et al., 2010 [72]: Participants are characterized as adolescents in this review despite that they were referred to as children in the original publication. The reason is that the youth were aged 13 to 19 years; Cimsir, E. and Akdogan, R., 2021 [55]: It contains 5 studies; only Study 5 characteristics are reported here because the first 4 studies are all about development of the emotional incest scale and its validation.

### 3.2. Risk of Bias and Certainty Assessment

As noted above, MMAT criteria ratings were used to assess study risk of bias, rigor, and quality. For the 13 qualitative studies and one mixed methods study (which reported primarily qualitative data), approximately 86% explicitly described appropriate research questions or objectives, data collection methods, findings, and interpretation, whereas the remaining 14% provided some description or narrative to substantively meet the criteria. Nearly 86% had medium to high coherence between data, analysis, and interpretation for at least one study aim or objective. Given these high MMAT criteria ratings, it was concluded that the risk of bias was low, and the study quality was high for the qualitative/mixed studies included in this review. Across the 81 quantitative articles, about 67% used appropriate measures for intervention/exposure and outcomes, nearly 73% specified at least one inclusion or exclusion criteria related to the target population, and 65% met at least one of the criteria for analyses that accounted for confounders. The authors relied on substantive comments rather than calculated scores, as encouraged by tool developers. We conclude that our review reflects a minimum risk of bias as it comprehensively incorporates the study literature.

### 3.3. Measurement Pattern Synthesis

#### 3.3.1. Parentification Measures

Original measure names and descriptions as well as study-specific modifications and psychometric properties are presented in Table 4 for 11 unique measures used by two or more studies (10 measures were only used by one study; not shown). Measures are organized by frequency of use (alphabetically for tied measures) and studies within a measure are alphabetically ordered by first author’s last name. Studies using more than one measure appear more than once in Table 4. Reliabilities were generally acceptable (α: 0.70 to 0.95) with a few exceptions. Of the 78 total entries across 21 measures, 53 (67.9%) made modifications to the original measures. Common modifications to original measures included using select subscales, reducing the number of items, or adopting peer-reviewed translations. Languages in which measures have been administered include Dutch, English, French, German, Hebrew, Polish, and Russian.

The most frequently used measure (*n* = 18) was the *Parentification Questionnaire (PQ)*. It was first developed by Sessions and first presented in its 42-item format with psychometric properties in a thesis [115]. The original measure includes three subscales assessing instrumental and emotional parentification and perceived unfairness. A few studies used only one or two of the original subscales. Reliabilities ranged from 0.61 to 0.92 with four studies having Cronbach alphas below 0.70 and typically included younger participants (adolescents or young adults).

The second most commonly used measure (*n* = 14), the *Parentification Inventory (PI)*, was developed by Hooper [116]. The 22-item scale includes three subscales: parent-focused parentification (PFP), sibling-focused parentification (SFP), and perceived benefits of parentification (PBP). Alphas ranged from 0.58 to 0.89 with the SFP characterized by lower reliability. The third most often used measure (*n* = 10) was the *Parentification Scale (PS),* which was developed by Mika and colleagues [117]. This 30-item scale includes four subscales: (1) child is parent to parent; (2) child is spouse to parent; (3) child parents siblings; and (4) child acts in all three ways. Alphas ranged from 0.57 to 0.95.

A less commonly used measure (*n* = 7), the *Filial Responsibility Scale, Adult Version (FRS)*, developed by Jurkovic, Thirkield, and Morrell [118], includes 60 items to assess six subscales: Past and Current Instrumental Caregiving, Past and Current Emotional Caregiving, and Past and Current Unfairness. Alphas ranged from 0.74 to 0.94. The *Parentification Questionnaire for Youth (PQ-Y)*, developed by Godsall and Jurkovic [119] and modified from the adult version [120], was used by five studies. It includes 20 items that assess emotional and instrumental parentification without explicit subscales. Alphas ranged from 0.64 to 0.80. Six measures detailed in Table 4 were used by three (*Family Structure Survey* and *Inadequate Boundaries Questionnaire*) or two studies (*Child Caretaking Scale, Childhood Questionnaire, Parent–Child Boundaries Scale III,* and *Relationship with Parents Scale*).

Several parentification measurement patterns were observed. First, studies typically focused on role-based (e.g., sibling-focused) or function-based (e.g., emotional) parentification, and it is not common that studies assess all these dimensions of parentification. Second, there is a dearth of measures designed to capture the parentification of youths either assessing their current experiences of parentification or retrospective parentification accounts of earlier experiences. Studies that included youth or youth measures reported lower reliabilities, suggesting effort should be made to develop a more reliable measure for youth. Third, most measures assessed adult retrospective accounts of parentification during childhood. Only one of seven studies using the *Filial Responsibility Scale, Adult Version* used *current* subscales, whereas the others focused on *past* subscales.

**Table 4:** Parentification Measures—Descriptions, Modifications, and Reliabilities.

Measure Name & Description	Literature	Participant Group	Modifications	Alpha	Sample Context
**The Parentification Questionnaire (PQ)** [115]: 42 Items, self-report, rate from 1 (usually not true/strongly disagree) to 5 (usually true/strongly agree) or true/false; 3 subscales: emotional and instrumental parentification, and perceived unfairness [17]: says only instrumental and emotional) (one article said that there are no official subscales). Includes emotional, expressive, and physical statements. The questions were summed.	Arellano et al., 2018 [40]	Adults	Use continuous PQ subscale scores and dichotomized subscale scores (subscales dichotomized into never/rarely experienced as scored less than 2 vs. some/repeated experiences as scored 2 and above); This study also used Parentification Inventory	Instrumental: 0.85 Emotional: 0.85 Unfairness: 0.92	College students
PQ	Carroll and Robinson, 2000 [49]	Adults	None	None reported	College students; have/do not have alcoholic and/or workaholic parents
PQ	Castro et al., 2004 [50]	Adults	None	None reported	College students in clinical and counseling psychology graduate programs
PQ	Hooper et al., 2008 [17]	Adults	None	Emotional: 0.75 Instrumental: 0.80	College students
PQ	Jankowski and Hooper, 2014 [67]	Adults	Only used the perceived unfairness scale (PQ-UN)	Perceived Unfairness: 0.89	College students
PQ	Jankowski et al., 2013 [68]	Adults	None	Instrumental: 0.84 Emotional: 0.84 Perceived Unfairness: 0.90	College students
PQ	McGauran et al., 2019 [76]	Adults	None	0.83	Offender/non-offender samples; all white
PQ	Oznobishin and Kurman, 2009 [85]	**Study 1:** Adults **Study 2:** Adolescents	**Study 1:** Combined PQ with Parent–Child Role Reversal Scale from the Family Structure Survey [121] (49 items total); Two factors emerged: **child dominance** (16 items) and **family support** (9 items) **Study 2: Child dominance** (27 items; from Study 1 and other role reversal questionnaires assessing emotional and instrumental) **Both studies:** translated into Hebrew and Russian	**Study 1: child dominance:** 0.80 (immigrants); 0.85 (Israeli- born) **Study 2: child dominance:** 0.89 (immigrants); 0.91 (Israeli-born)	College students (Study 1) and high school students (Study 2); both studies include immigrant or Israeli-born
PQ	Rogers and Lowrie, 2016 [90]	Adults	Only used 21 items without specification what items they selected		Age ranged from 19 to 92 years old; 82.4% Caucasian
PQ	Titzmann, 2012 [98]	Adolescents and their mothers	Translated into Russian and German; Used 2 subscales: Emotional and instrumental [17,110]; Items based on PQ and Parentification scale [117,120]; Emotional and Instrumental: mean of five items rated on a six-point scale	Emotional: 0.70 Instrumental: 0.69	Ethnic (185) and 197 native German families
PQ	Titzmann and Gniewosz, 2018 [99]	Adolescents and their mothers	Only assessed instrumental; mean of 5 items; 6 pt Likert scale; based on Parentification Scale [117] too	Instrumental: 0.69	Ethnic German immigrant mother-adolescent dyads from the former Soviet Union
PQ	Van der Mijl and Vingerhoets, 2017 [103]	Adults	None	0.84	College students
PQ	Wei et al., 2020 [114]	Young adults	Three items were used from each subscale	Instrumental: 0.61 Emotional: 0.66 Perceived fairness: 0.77	Junior and senior high school students
PQ	Wells and Jones, 1998 [108]	Adults	None	None reported	College students
PQ	Wells and Jones, 2000 [109]	Adults	None	None reported	College students
PQ	Wells et al., 1999 [107]	Adults	None	None reported	College students
PQ	Williams and Francis, 2010 [110]	Adults	None	None reported	College students
PQ	Yew et al., 2017 [112]	Adults	None	0.79	College students in clinical and nonclinical academic programs
**The Parentification Inventory (PI)** [122,123]: adult self-report, 22 items on a 1 (never true) to 5 (always true) response scale; three subscales include parent-focused parentification (PFP), sibling-focused parentification (SFP), and perceived benefits of parentification (PBP). Scales are summed and averaged	Arellano et al., 2018 [40]	Adults	NA as an option on PI; Only parent-focused and sibling-focused parentification subscales; subscales were dichotomized (rarely or never experienced vs. those who had some level of or repeated experience); Also used the PQ [118]	Parent-focused: 0.78 Sibling-focused: 0.65	College students
PI	Beffel and Nuttall, 2020 [42]	Adults	None	PFP: 0.83 SFP: 0.79 PBP: 0.88	College students; typically developing (TD) who reported having a sibling with Autism Spectrum Disorder (ASD)
PI	Borchet and Lewandowska-Walter, 2017 [43]	Late adolescents	Experimental version of the Polish adaptation	PFP: 0.80 SFP: 0.58 PBP: 0.81	Individuals
PI	Borchet et al., 2016 [46]	Late adolescents and their parents	Used Kwestionariusz Parentyfikacji (KP)- experimental version of the Polish adaptation of the PI	PFP: 0.75 SFP: 0.60 PBP: 0.89	Family triads; families recruited through students
PI	Borchet, Lewandowska-Walter, Połomski, Peplińska, and Hooper, 2020 [45]	Late adolescents	Used a Polish adaptation of PI; used the perceived benefits of parentification subscale only	PBP: 0.77	School students
PI	Burton et al., 2018 [48]	Early adolescents	None	PFP: 0.82 SFP: 0.63 PBP: 0.85	Middle and high school students
PI	Chen et al., 2018 [53]	Adolescents (18–24)	Used 19-item three-factor structure of the scale: household responsibility, perceived benefits, and spousal parentification	Household responsibility: 0.87; Perceived benefits: 0.84; Spousal parentification: 0.77	Transitional-aged youth
PI	Cimsir and Akdogan, 2021 [55]	Adults	Only used PFP subscale; adapted the PI into Turkish: Factor structure varied slightly for subscales (Turkish culture normalizing parentification)	PFP: 0.84	College students
PI	Hooper et al., 2015 [66]	Adults	None	Total Sample: PFP: 0.79 SFP: 0.58 PBP: 0.80	College students
PI	Hooper, Wallace et al., 2012 [3]	Adults	None	PFP: 0.83 SFP: 0.80 PBP: 0.80 Black: PFP: 0.83 SFP: 0.76 PBP: 0.80 White: PFP: 0.85 SFP: 0.79 PBP: 0.81	College students
PI	Murrin et al., 2021 [78]	Adults	Only mentions PFP and SFP; likely only used 19 items	PFP: 0.83 SFP: 0.79	College students; typically developing (TD) who reported having a sibling with Autism Spectrum Disorder (ASD)
PI	Nuttall et al., 2018 [81]	Adults	None	PFP: 0.83 SFP: 0.79 PBP: 0.88	College students; TD who reported having a sibling with ASD
PI	Tomeny, Barry, and Fair, 2017 [101]	Adults	Only used PFP and SFP	Each of the PI subscales ranged from 0.64 to 0.88	College students; TD who reported having a sibling with ASD
PI	Tomeny, Barry, Fair, and Riley, 2017 [100]	Adults	1 item removed from SFP to improve internal consistency	SFP: 0.72–0.88	TD who reported having a sibling with ASD
**Parentification Scale** [117]: adult self-report, 30 items on a 1 (never or does not apply) to 5 (very often) response scale; subscales where child is functioning (1) as a parent to parent(s), (2) as a spouse to parents, (3) as a parent to sibling(s), and (4) in ways which transcend these subtypes. Includes particular subtypes (e.g., consoler, adviser, confidant, or peacemaker). Questions asked how often behavior occurred before the age of 14 and how often it occurred between the ages of 14 and 16. Differential weights were assigned to the questions depending on the age and physical/emotional burden	Fitzgerald et al., 2008 [58]	Adults	Only used 3 subscales (acting as a parent to parent, spouse to parent, and parent to siblings); Items were summed	Parent to parent: 0.76 Spouse to parent: 0.78 Parent sibling: 0.86	College students
PS	Perrin et al., 2013 [87]	Adults	Only used 1 item from Parental Role with Parents subscale; The final scale included 17 items; Items were also drawn from Parent-Child Boundaries Scale III [124], Family Structure Survey [121], and Filial Responsibility Scale-Adult; Item sets asked about mothers and fathers	17-item Parentification Scale created; Mothers: 0.94 Fathers: 0.93	College students
PS	Sang et al., 2014 [20]	Adolescents and their mothers	Only used 3 of 4 subscales: (a) spousal role vis-a-vis parents (8 items); (b) parental role vis-a-vis parents (6 items); (c) parental role vis-a-vis siblings (12 items)	Range: 0.84 to 0.92	Black and Hispanic mother; HIV-negative daughter; low-income inner-city sample recruited in agencies that provided services to HIV-infected women; victims of intimate partner violence; and those in substance use recovery
PS	Shin and Hecht, 2012 [94]	Adolescents	Two items from Parentification scale operationalized problem-solving parentification; 5 point scale, but went from strongly disagree to strongly agree; used FRS as well	None reported	Mexican-heritage; middle school students; use Wave 4–6 only
PS	Stein et al., 1999 [95]	Adolescents and one of their parents	Parental role with siblings was not used because not applicable to many of the study participants	Adult role-taking: 0.77 Spousal role: 0.75 Parental role: 0.67	Non-infected adolescents of parents with AIDS
PS	Stein et al., 2007 [19]	Adolescents and one of their parents	Parental role with siblings was not used; many participants did not have siblings; used the mean	None reported	Children with HIV/AIDS-infected mothers
PS	Titzmann, 2012 [98]	Adolescents and their mothers	Translated into Russian and German; items based on PS and PQ [117,120] (Emotional and Instrumental: mean of five items rated on a six-point scale; selected individual items from subscales)	Emotional: 0.70 Instrumental: 0.69	Ethnic (185) and 197 native German families
PS	Titzmann and Gniewosz, 2018 [99]	Adolescents and their mothers	Only assessed instrumental; mean of 5 items; 6 pt Likert scale; based on PQ [120] too	Instrumental: 0.69	Ethnic German immigrant mother-adolescent dyads from the former Soviet Union
PS	Tompkins, 2007 [102]	Adults and Adolescents	3 items deleted of the parental role to parent scale due to inadequate reliability (involved fathers or illness)	Mother/Child: Spousal role: 0.76 Parental role to siblings: 0.95/0.86 Non-specific adult responsibilities: 0.78/0.65 Parental role to parent: 0.69/0.71	Children with HIV/AIDS-infected mother (*n* = 23) vs. children with HIV-seronegative mother (*n* = 20)
PS	Walsh et al., 2006 [105]	Adolescents	Translated into Hebrew and Russian; Deleted 3 items	Hebrew/Russian: Spousification: 0.73/0.84 Parental role for parents: 0.57/0.79 Parental role for siblings: 0.80/0.87 Nonspecific adult role taking: 0.82/0.78.	Study 1: Immigrants from former Soviet Union in Israel vs. Israel born; Study 2: Immigrants from former Soviet Union in Israel
**Filial Responsibility Scale, Adult Version (FRS)** [118]: adult self-report; 60 items on a 1 (strongly disagree) to 5 (strongly agree) response scale; 6 subscales: Past Instrumental Caregiving, Past Emotional Caregiving, Past Unfairness, Current Instrumental Caregiving, Current Emotional Caregiving, Current Unfairness	Cho and Lee, 2019 [54]	Adults	Only used past subscales	Instrumental: 0.74 Emotional: 0.78 Unfairness: 0.87	College students
FRS	Madden and Shaffer, 2016 [75]	Adults	Only used past Emotional and past Instrumental subscales	Emotional: 0.82 Instrumental: 0.80	College students
FRS	Nuttall, Ballinger, Levendosky and Borkowski, 2021 [80]	Adults	Only used three subscales (past)	0.92	Majority of mother sample were non-White (78.4%) and unmarried (74%)
FRS	Nuttall et al., 2012 [82]	Adults	Only used three subscales (past); summed subscales	0.92	Community sample; high-risk, first-time adolescent and adult mothers
FRS	Nuttall et al., 2019 [84]	Adults, adolescents	Used past emotional and past instrumental only	Both: 0.78	Predominantly low-income and ethnic minorities; college students; psychosis-proneness sample
FRS	Perrin et al., 2013 [87]	Adults	Only used 4 items from Current Emotional Caregiving subscale (final scale created included 17 items); items written twice (one asked about mothers and one fathers); also used items from Parent- Child Boundaries Scale III [124], Family Structure Survey [121], and Parentification Scale [117]; never to almost always	17 item Parentification Scale created: Mothers: 0.94 Fathers: 0.93	College students
FRS	Shin and Hecht, 2012 [94]	Adolescents	Used two items from FRS operationalized adult parentification; used the Parentification Scale [117] as well	None reported	Mexican-heritage; middle school students; use Wave 4–6 only
**Parentification Questionnaire for Youth (PQ-Y)** [119]: 20 items, yes/no format, no official subscales, but some more emotional or instrumental in nature; modified version of the PQ-A [120]: items changed to present tense and 3rd grade vocab	Chen and Panebianco, 2020 [52]	Adolescents	Only used Emotional Parentification subscale; created instrumental parentification with 22 items from three other instruments	Kuder-Richardson reliability coefficient was 0.72	Middle and high school students with at least one parent with a chronic illness
PQ-Y	Fortin et al., 2011 [59]	Children and their mothers	Remove 5 items that measured the family’s recognition of the child’s parentification due to reduced instrument reliability	0.64 reported	Children exposed to domestic violence
PQ-Y	Godsall et al., 2004 [60]	Children and adolescents	Item total reduced to 20 (not appropriate for children, and did not meet item total correlation)	0.76 Cross-validation: 0.75	High-functioning/low-functioning children; students
PQ-Y	Hooper, Doehler et al., 2012 [65]	Adolescent and parent pairs	None	0.80	Rural community sample
PQ-Y	Van Loon et al., 2017 [104]	Adolescents and one parent	Translated into Dutch; no clear factor structure (emotional vs. instrumental) so used sum score	0.69	Adolescents living with a parent with mental health problems
**Family Structure Survey (FSS)** [121]: adult self-report; 50 items on a 1 (completely false) to 5 (completely true) response scale; adults’ recalled perceptions of their family interactions on four dimensions of dysfunctional family structure: parent-child over involvement, fear of separation, parent-child role reversal, and marital conflict; total scale scores are not used	King and Mallinckrodt, 2000 [71]	Adults	None	Parent-child role reversal: 0.74	College students; clinical/nonclinical samples
FSS	Oznobishin and Kurman, 2009 [85]	**Study 1:** Adults **Study 2:** Adolescents	**Study 1:** Combined PQ with Parent–Child Role Reversal Scale from the Family Structure Survey [121] (49 items total); Two factors emerged: **child dominance** (16 items) and **family support** (9 items) **Study 2: Child dominance** (27 items; from Study 1 and other role reversal questionnaires assessing emotional and instrumental) **Both studies:** translated into Hebrew and Russian	**Study 1: child dominance:** 0.80 (immigrants); 0.85 (Israeli- born) **Study 2: Child dominance:** 0.89 (immigrants); 0.91 (Israeli-born)	College students (Study 1) and high school students (Study 2); both studies include immigrant or Israeli-born
FSS	Perrin et al., 2013 [87]	Adults	Final scale created included 17 items; 3 items from the Parent-Child Role Reversal subscale; also used items from the Parent-Child Boundaries Scale III [124], Filial Responsibility scale and PS [117]; never to almost always	17 item Parentification Scale created: Mothers: 0.94 Fathers: 0.93	College students
**Inadequate Boundaries Questionnaire** [125]: adult self-report; 34 items on a 1 (almost never) to 5 (almost always) response scale for different types of boundary dissolution with their own mothers as children; dimensions include guilt induction, blurring of psychological boundaries, parentification (emotional and instrumental), triangulation, and the use of psychological control	Golan and Goldner, 2019 [61]	Adults	Made a guilt-psychological control scale and boundaries-parentification scale	Triangulation: 0.87 Blurring boundaries: 0.62 Parentification: 0.87 Boundaries-Parentification: 0.89	Young, first-time Jewish mothers of children aged 12–36 months
IBQ	Goldner et al., 2017 [62]	Adolescents		Parentification (both): 0.74	Middle school students
IBQ	Goldner et al., 2019 [63]	Adolescents	Completed the Parentification and the Enmeshment with the Mother subscales	Parentification: 0.74 Enmeshment: 0.69	Convenience sample; drawn from mid- to high-SES middle schools
**Child Caretaking Scale** [126]: child self-report; 30 items on a 1 (strongly disagree) to 5 (strongly agree) response scale; designed originally for children living with a mother experiencing psychiatric difficulties	Khafi et al., 2014 [70]	Children and their mothers	18 items were used to identified emotional (8 items) and instrumental (10 items) subscales	T1 Emotional: 0.59 T2 Emotional: 0.70 T1 Instrumental: 0.66 T2 Instrumental: 0.66	Sample overrepresents mothers with anxiety, affective, and/or substance use disorders; predominantly low-income
CCS	McMahon and Luthar, 2007 [77]	Children and their mothers	25 items used to define three dimensions of caretaking burden: responsibility to care for mother, responsibility for household chores, and responsibility to care for siblings	Care for mother: 0.63 Household chores: 0.61 Care for siblings: 0.75	Urban, low-income children living with biological mothers; includes mothers (a) with drug problem, (b) with psychiatric problem, or (c) none of the two
**Childhood Questionnaire (CQ)** [127]: adult self-report; first 14 years; mothers and fathers; 20 items (only including subscales) assess parent-child relationships; dimensions include Perceived Love, Control, Ambition and Role Reversal (4 item scale on SES, individual items on separation and divorce of parents, eventual death of either or both of the parents, and education and occupation of the parents during the subject’s childhood); 4 pt Likert scale from not true at all to absolutely true	Dragan and Hardt, 2016 [56]	Adults	None	None reported	Subjects all registered with a market research company
CQ	Schier et al., 2015 [91]	Adults	None	None reported	Internet survey; extraction and cross-validation samples
**Parent–Child Boundaries Scale III (PBS-III)** [124]: is a 53-item self-report measure. Measures general parentification (no empirically supported subscales of emotional or instrumental parentification, but have items indicative of these); 5-point Likert-type scale ranging from 1 (never) to 5 (almost always)	Perrin et al., 2013 [87]	Adults	17 items were also drawn from the Filial Responsibility Scale—Adult, Family Structure Survey [121], and Parentification Scale [117]; items were asked about mothers and fathers)	17 item Parentification Scale created: Mothers: 0.94 Fathers: 0.93	College students
PBS-III	Baggett et al., 2015 [41]	Adults	Only used 6 items; items combined to create global parentification scale	0.89	College students
**Relationship with Parents Scale (RPS)** [128]: A 42-item (21 items: mother, 21 items: father) self-report retrospective measure of parent-child role reversal; 5-point scale 1 (strongly disagree)- 5 (strongly agree)	Abraham and Stein, 2013 [39]	Adults	Only used 21 items (Mother version: reflected mothers using guilt to elicit nurturing from them, demanding their attention or company, and their perception of their mother’s competence as a parent)	0.93	Emerging adults who have a mother with/without mental illness and poor psychological adjustment
RPS	Katz et al., 2009 [69]	Adults	None	Fathers: 0.89 Mothers: 0.92	College students; grew up in an intact family with one mother and one father
**Maastricht Parentification Scale** [129]: self-report; 22 items on a 1 (completely disagree) to 4 (completely agree) response scale for parents reporting on both their own parenting and the parenting of their partner; low scores are indicative of psychological autonomy, whereas high scores are indicative of psychological control; 6 subscales of parentification: emotional care parents, buffer between parents, household care family, financial care family, instrumental care siblings, emotional care siblings	Boumans and Dorant, 2018 [47]	Adults	None	Emotional care parents: 0.78 Buffer between parents: 0.71 Household care family: 0.76 Financial care family: 0.68 Instrumental care siblings: 0.76 Emotional care siblings: 0.71	College students; carers/non-carers
**Parentification Questionnaire for Youth (PQY)** [44]: self-report, 26 items on a 1 (never true) to 5 (always true) response scale, four subscales: emotional parentification toward parents, instrumental parentification toward parents, sense of injustice, and satisfaction with the role; and two subscales for adolescents who have siblings: instrumental parentification toward siblings and emotional parentification toward siblings. scores are calculated as the mean of the ratings for the subscale items	Borchet et al., 2021 [8]	Adolescents	None	0.70 to 0.80	Polish sample; majority self-identified college students
**Perceived Parental Rearing Behavior Questionnaire** [130]: adult self-report; 30 items on a 1 (not at all) to 5 (all the time) response scale; dimensions include transmission, affection, punishing, over-involvement/protection	Hoffman and Shrira, 2019 [64]	Adults	Included 20 items; conceptualizes transmission as role reversal; translated to English	Role reversal: 0.71 Affection: 0.88 Punishing: 0.66 Overinvolvement: 0.70	Community sample; Jewish parents of European origin born before 1945 and their offspring born after 1945; parents were alive during World War II and either Holocaust survivors or had no Holocaust background
**Triangulation** [131]: 45 items, 1 (totally disagree) to 3 (totally agree); 3 dimensions: cross-generation coalition, scapegoating, parentification	Wang et al., 2017 [106]	Children	22 items were removed due to length consideration, or lack of association with other items as determined by exploratory factor analysis	Coalition: 0.79 Scapegoating: 0.75 Parentification: 0.72	Two elementary school and three high school students

#### 3.3.2. Outcome Measures

Measure names, descriptions, study-specific modifications, and psychometric properties are presented in Table 5 for the constructs studied more than twice. Close to 30% of the studies are not included in Table 5 because they included constructs studied no more than twice. Broadly, the constructs excluded from Table 5 can be grouped into positive life outcomes, family relationships, and clinically relevant outcomes. Constructs in Table 5 are ordered based on the frequency of being studied. Within each construct, measures are ordered from the highest to the lowest frequency. Note that studies may appear multiple times when they investigated more than one construct or used more than one measure for one construct.

The most frequently studied construct was depression (*n* = 21), followed by internalizing and/or externalizing problems (*n =* 12). These constructs were all measured with well-validated instruments, and depression had good reliability. However, over half of the studies on internalizing and/or externalizing problems failed to report reliability for their specific studies.

Other negative outcomes include psychological/emotional distress which was often analyzed as a latent variable (*n* = 8), substance use (*n* = 10), antisocial behavior (*n* = 3), and risky sex (*n* = 3). The last three constructs were behavior-based measures and often measured by indicating the presence or frequency of the behavior [132,133]. Except for three studies that used the same instrument for substance use [3,65,67], no two studies used the same measures for antisocial behavior, risky sex, or substance use. Positive outcomes include self-esteem (*n* = 5), satisfaction with life (*n* = 3), and efficacy (*n* = 3), and they were measured similarly across studies with good reliability.

Several patterns were observed for outcome measures. First, outcome constructs were routinely assessed with well-validated instruments and reported good reliability. Second, only very few studies examined positive outcomes. This focus on adverse outcomes neglects the potential positive outcomes of parentification. Third, when constructs were only studied a couple of times, there is not ample evidence to conclude whether and how these constructs are related to parentification. Fourth, studies that focused on the same constructs only a few times were often conducted by the same researcher groups, which may suggest the limited research scope and lack of attempts to integrate individual research into the broader literature of parentification. 

**Table 5:** Outcome Measures—Descriptions and Psychometric Properties.

Outcome Construct	Description	Literature	Sample Context	Modifications	Alpha	Associations with Parentification
**Depression**	The Beck Depression Inventory II (BDI-II) [134]: 21 questions, self-report, rate on a 4-point scale from 0 (absence of symptoms) to 3 (severe presence of symptoms) for depressive symptoms. Items are summed	Arellano et al., 2018 [40]	College students	Used continuous score and the dichotomized score for BDI-II (20 and above high; rest low)	0.91	PFP+, SFP ns, EP+, IP+, Unfairness+
		Carroll and Robinson, 2000 [49]	College students; have/do not have alcoholic and/or workaholic parents	None	None stated	Overall no direct test
		Hooper, Doehler et al., 2012 [65]	Rural community sample	Adolescent self-report; parent self-report	Parent report: 0.94; Adolescent: 0.92	Overall ns
		Hooper et al., 2015 [66]	College students		0.92	PFP +, SFP +, PBP −
		Hooper, Wallace et al., 2012 [3]	College students	None	Overall: 0.91 Black: 0.90 White: 0.92	White: PFP ns, SFP ns, PBP −; Black: PFP +, SFP ns, PBP −
		Jankowski et al., 2013 [68]	College students	Used it together with GSI from BSI to create a latent variable of mental health symptoms	0.92	Overall +
		Prussien et al., 2018 [88]	Mothers with children diagnosed with cancer	Mother self-reported	0.93	Emotional caregiving ns
	The Center for Epidemiologic Studies Depression Scale (CES-D) [135]: 20 items, self-report, rate on a 4-point scale from 0 (rarely or not at all) to 3 (most of the time). Items are summed.	Chen and Panebianco, 2020 [52]	Middle and high school students with at least one parent with a chronic illness	None	0.90	EP +, IP ns
		Cho and Lee, 2019 [54]	College students	None	0.91	EP ns, IP ns, Unfairness +
		Katz et al., 2009 [69]	College students; grew up in an intact family with one mother and one father	None	0.90	Role reversal ns
		Murrin et al., 2021 [78]	College students; typically developing (TD) who reported having a sibling with Autism Spectrum Disorder (ASD)	None	0.87	PFP ns, SFP ns
		Nebbitt and Lombe, 2010 [79]	African Americans living in urban, public housing developments	Rated frequency of symptoms in terms of days per week from 0 (less than 1 day) to 3 (5–7 days)	0.88	Household contribution—
		Wang et al., 2017 [106]	Two elementary school and three high school students	None	0.86	Coalition +, scapegoating +, Parentification −
	The Children’s Depression Inventory (CDI) [136]: 27-item, self-report, depressed mood. Rate from 0 (once in a while) to 2 (all the time)	Fortin et al., 2011 [59]	Children exposed to domestic violence	Used with the anxiety construct to form a latent variable internalizing problems	0.84	Overall +
		Khafi et al., 2014 [70]	Sample overrepresents mothers with anxiety, affective, and/or substance use disorders; predominantly low-income		T1: 0.81 T2: 0.87	T1 IP and T2 dep ns, T1 EP and T2 dep +
		Rodriguez and Margolin, 2018 [89]	Adolescents in active-duty military families	Omitted suicidal ideation item due to ethical and reporting concerns	0.83	IP −, EP ns, Observed emotional validation −
		Tompkins, 2007 [102]	Children with HIV/AIDS-infected mother (23) vs. children with HIV-seronegative mother (20)		Only have range for all measures (0.70–0.89)	Mother report child’s parentification—(correlate only)
	The Brief Symptom Inventory-18 (BSI-18) [137]: 18 items, self-report, rate on a 5-point Likert scale from 0 (not at all) to 4 (very much). But Depression subscale only has 6 items. Mean score of 6 items for depression	Hoffman and Shrira, 2019 [64]	Community sample; Jewish parents of European origin born before 1945 and their offspring born after 1945; parents were alive during World War II and either Holocaust survivors or had no Holocaust background	Hebrew version; Parent completed interview and offspring completed questionnaires including depression subscale derived from BSI-18	0.86	Role reversal +
	Positive and Negative Affect Schedule for Children (PANAS-C) [138]: 30 items, self-report, rate on a 5-point scale form 1 (very slightly or not at all) to 5 (extremely). 15 items each for the positive affect and negative affect scales	Burton et al., 2018 [48]	Middle and high school students	Positive affective is used as wellbeing; and negative affective is used as depressive symptoms	NA: 0.90 PA: 0.91	PFP +, SFP ns, PBP −
	The Symptom Checklist-27-plus (SCL-27-plus) [139] 27 items, self-report, rate on a 5 point scale how often (‘‘never’’ to ‘‘very often’’) symptoms occur in past 2 weeks. 5 dimensions in total	Schier et al., 2015 [91]	Internet survey; extraction and cross-validation samples	Used depression (5 items) only	None stated	Maternal EP +, Paternal EP +,
	The Weinberger Adjustment Inventory (WAI) [140]: 62-item, self-report, rate on a 5-point Likert scale from 1 (false) to 5 (true). Distress and Restraint as 2 primary dimensions, each defined with 4 subscales	Williams and Francis, 2010 [110]	College students	Only used depression and happiness subscales, each contains contain 7 items	None stated	Overall +
**Distress**	The Brief Symptom Inventory (BSI) [141]: 53 items, self-report, rate on a 5-point Likert scale from 0 (not at all) to 4 (extremely). Three broad global indices are global severity index, positive symptom distress index and positive symptom total.	Hooper et al., 2008 [17]	College students	Used just the positive symptom total to capture “distress”	0.96	EP +, IP ns
		Jankowski et al., 2013 [68]	College students	Summed all items and divided by 53 to get GSI; Used it together with BDI to create a latent variable mental health symptom	GSI: 0.97	Overall +
		Lester et al., 2010 [72]	Adolescent with HIV/AIDS-infected mothers, or adolescent of neighborhood control mothers	Child report; Used depression and anxiety subscale to form a latent variable for emotional distress	Depression: 0.81 anxiety: 81	Role reversal ns
		Oznobishin and Kurman, 2009 [85]	College students (Study 1) and high school students (Study 2); both studies include immigrant or Israeli-born	Sum score; called it “psychological distress”	Immigrant: 0.96 Israel born: 0.95	Child dominance ns, Language brokerage ns
		Stein et al., 1999 [95]	Non-infected adolescents of parents with AIDS	Used mean scores from each of three subscales depression, anxiety, and phobic anxiety as indicators for latent variable internalized emotional distress	0.79, 0.79, 0.80, respectively	PFP ns, SFP ns, Non-specific adult role ns.
		Stein et al., 2007 [19]	Children with HIV/AIDS-infected mothers	Used mean scores from each of three subscales depression, anxiety, and phobic anxiety as indicators for latent variable emotional distress; Assessment time window: during the past week, including today; Assessed at baseline and at year 6	None stated	Overall parentification and distress 6 yrs later ns
	The Depression, Anxiety, and Stress Scale 21 (DASS-21): 21 items, self-report, rate on a 4-point scale from 0 (not at all) to 3 (very much/most of the time). Sum scores multiplied by 2 [142] to make it comparable to the 42-item DASS	Tomeny, Barry, and Fair, 2017 [101]	College students; typically developing (TD) who reported having a sibling with Autism Spectrum Disorder (ASD)	Used overall score as “distress”	0.84 to 0.91 for subscales, but did not report overall scale	PFP ns, SFP ns
	The Profile of Mood States (POMS) [143]: daily diary studies commonly used measure for stress and psychological well-being, rate on a 5-point scale from 1 (not at all) to 5 (extremely) to indicate the extent to which they felt distress with 7 items	Telzer and Fuligni, 2009 [97]	High school students; ethnically diverse sample of adolescents from predominantly Latin American, Asian, and European backgrounds	Each evening during the 2-week period, adolescents’ daily mood assessed	0.76 and 0.94 for daily level and individual level	Perceived demand +, Role fulfilment −
**Internalizing and externalizing problems**	Child behavior Checklist/teacher report form (CBCL/TRF) [144]: 113 items, teacher rating, rate on a 3 point scale form 0 (not true) to 2 (very or often true). 8 subscale symptoms: internalizing problems—withdrawn, somatic complaints, anxiety and depression. Externalizing problems—aggressive behavior, and delinquent behaviors	Champion et al., 2009 [51]	Mother with/without history of depression from urban area	Used only the depression-anxiety symptoms, and the social competence; Mother reported on adolescents’ symptoms	None stated	EP +, IP ns
		Macfie et al., 2005 [73]	Rural families; waves of data collected on families	Used four composite scores: internalizing, externalizing, attention problems, and social problems; Teacher report child behavior when child is 5 yr 10 mos	None stated	Paternal role reversal ns, maternal role reversal ns
		Nuttall, Valentino, Cummings, and Davies, 2021 [83]	Data collected when children were in kindergarten (Wave 1), first grade (Wave 2), and second grade (Wave 3)	Mother reported, and father reported at Wave 1–3	Ext: 0.87–0.90 Int: 0.84–0.88	
		Peris et al., 2008 [86]	Family triads, longitudinal design		None stated	Maternal EP and EXT/INT+
		Prussien et al., 2018 [88]	Mothers with children diagnosed with cancer	Mother reported; only look at anxious/depressed symptom subscale	T1: 0.74 T3: 0.86	Emotional caregiving ns boys, + girls
	Youth Self Report (YSR) [145]: 112 items, self-report, rate from 0 (not true) to 2 (Very true or often true). 8 subscale symptoms: internalizing problems—withdrawn, somatic complaints, anxiety and depression. Externalizing problems—aggressive behavior, and delinquent behaviors	Champion et al., 2009 [51]	Mother with/without history of depression from urban area	Used only the depression-anxiety symptoms, and the social competence	None stated	EP+, IP ns
		Peris et al., 2008 [86]	Family triads, longitudinal design		None stated	Maternal EP and EXT/INT+
		Shaffer and Egeland, 2011 [92]	Mother of low socioeconomic status recruited through a public health clinic for prenatal care	Internalizing and externalizing symptom were self-reported at age 16 T scores were used	Externalizing: 0.93. Internalizing: 0.91	For both EXT and INT: Childhood BD ns, Adolescent BD + (control for childhood BD)
		Van Loon et al., 2017 [104]	Adolescents living with a parent with mental health problems	EXT, INT measured at T1 and T2 one year apart	Ext: 0.82 at T1; 0.83 at T2; INT: 0.87 at T1; and 0.89 at T2	Overall +
		Wang et al., 2017 [106]	Two elementary school and three high school students	Only used the 17-item aggression subscale	None reported	Coalition ns, Scapegoating +, Parentification −
	The Behavior Assessment System for Children (BASC) [146]: 5 separate rating forms, including a teacher rating scale (TRS), a parent rating scale (PRS) a self-report of personality (SRP), student observation system (SOS) and a structured developmental history (SDH). The full measure has 105–165 items and uses 4-point scale from 0 (never) to 3 (almost always	Khafi et al., 2014 [70]	Sample overrepresents mothers with anxiety, affective, and/or substance use disorders; predominantly low-income	Mothers completed PRS for the Child (ages 8–11) or Adolescent (ages 12–18 version depending on the child’s age	Child externalizing: child form 0.93 and adolescent form 0.96 and 0.94 at T1 and T2	T1 EP for T2 EXT: Blacks ns, Whites +; T1 IP for T2 EXT ns
		McMahon and Luthar, 2007 [77]	Urban, low-income children living with biological mothers; includes mothers (a) with drug problem, (b) with psychiatric problem, or (c) none of the two	Parent report using PRS; and child report using the SRP child report INT, school maladjustment and social competence; mother report INT, EXT and social competence of the child	None stated	Linear and quadratic term of care for mother +
	The Infant-Toddler Social and Emotional Assessment (ITSEA) [147]: parent report, include a scale for externalizing problems (conceptualized as aggression, defiance, negative emotional reactivity and high activity)	Nuttall et al., 2012 [82]	Community sample; high-risk, first-time adolescent and adult mothers	Mother report child’s externalizing problems at 36 month of age	none stated	Overall +
	The Strengths and Difficulties Questionnaire (SDQ) [148]: 25-item, rate on a 3-point ordinal scale (0 = not true, 2 = certainly true)	Zvara et al., 2018 [113]	Rural, low-income families	Teacher rated version of the SDQ for children’s internalizing, externalizing symptoms, and peer problems at Grade 1	Internalizing: 0.80 Externalizing: 0.84 Peer problem: 0.73	INT: Role confusion + EXT: role confusion ns
**Anti-social Behavior**	10 items measuring antisocial behavior in the last 12 months at baseline and at follow-up. These items have been used in other national studies (e.g., The National Longitudinal Study of Adolescent to Adult Health). Participants responded on 5-point scale (0 = never; 1 = once, 2 = twice; 3 = 3 or 4 times; and 4 = 5 or more). Scores on each item were dichotomized then summed to a variety score	Chen et al., 2018 [53]	Transitional-aged youth		Baseline: 0.75 1-year follow up: 0.67	PFP ns, SFP ns, PBP −
	The Antisocial Behaviour Questionnaire (ABQ) [149]: 10 items, self-reports, rate on a 5-point scale form 1 (never) to 5 (always), sum score	McGauran et al., 2019 [76]	Offender/non-offender samples; all white	None	0.86	Overall ns
	The Conduct Disorder scale [150], 27 items, self-report about past 3 months. Rated on 1 (no) and 2 (yes) and sum items for score. Have three scales: aggression (5 items), criminal behavior (14 items) and rebellious behavior (3 items)	Stein et al., 1999 [95]	Non-infected adolescents of parents with AIDS	Excluded five items and used only 22 items; Used the three subscale to form a latent variable called conduct problem	0.78 for 27 item; 0.78 for 22-item	Parental role +
**Substance use**	The Alcohol Use Disorders Identification Test (AUDIT) [151]: 10 items, self-report, rate on a 5-point Likert scale from 0 (never) to 4 (daily or almost daily). Sum score	Hooper, Doehler, et al., 2012 [65]	Rural community sample	Parent self-report	None stated	Overall ns
		Hooper, Wallace, et al., 2012 [3]	College students	None	Total: 0.78 Black: 0.70 White: 0.79	PFP ns, SFP ns, PBP ns
		Jankowski and Hooper, 2014 [67]	College students	None	0.78	Unfairness +
	CAGE questionnaire [152]: The name CAGE derives from the four items: “Cut back on drinking”, “Annoyance at criticisms about drinking”, “Feeling Guilty about drinking” and “Using alcohol as an Eye opener”. All items require “yes” or “no” answers	Dragan and Hardt, 2016 [56]	Subjects all registered with a market research company	The item “Guilt” was excluded from the analysis due to the excessive difference between the samples of Poles and Germans; A binary variable “problematic alcohol use” was set to “1” whenever at least one of the three items was endorsed	None stated	Paternal role reversal ns
	One item only, derived from the Youth Risk Behavior Surveillance System (Centers for Disease Control and Prevention), “During the past 30 days, on how many days did you have at least one drink of alcohol?’’. Rate on a scale from 0 (0 days) to 7 (all 30 days)	Hooper, Doehler, et al., 2012 [65]	Rural community sample	Adolescent self-report	None stated	Overall ns
	3 items, including the number of cigarettes smoked per day, total frequency of drinks of any form, and quantity of alcohol use on the day of alcohol consumptions over the past 3 months	Stein et al., 2007 [19]	Children with HIV/AIDS-infected mothers	Used as latent variable	N/A	Overall predict use 6 yrs later −
	3 frequency indicators showing use of alcohol, hard drugs, and marijuana within the past 90 days	Lester et al., 2010 [72]	Adolescent with HIV/AIDS-infected mothers, or adolescent of neighborhood control mothers	Formed a latent variable with 3 indicators	N/A	Role reversal −
	3 primary indicators, alcohol frequency, marijuana frequency, and drug problems (sum of 5 items (no = 1, yes = 2) that assessed whether they had any problems with drug such as feelings of dependency or withdrawal symptoms) in past 3 months	Stein et al., 1999 [95]	Non-infected adolescents of parents with AIDS	Used to form a latent variable	None stated	Parental role +, Spousal role ns,
	self-report regarding lifetime use: Six response options ranged from 0 to 7 or more times. Recent use of substances: Six response options ranged from 0 days to 20–30 days	Sullivan et al., 2018 [96]	Middle and high school students	Only used the 4 most endorsed substances: (a) a whole cigarette, (b) one full drink of alcohol, (c) marijuana, and (d)inhalants	None stated	High parentification class have lower prob to be polysubstance user than in abstained class
	2 items, whether they ever use alcohol or marijuana	Sang et al., 2014 [20]	African American and Hispanic mother; HIV-negative daughter; low-income inner-city, recruited in agencies that provided services to HIV-infected women; victims of intimate partner violence, and those in substance use recovery	Lifetime use of substance; separated them for analyses	N/A	Overall ns, Spousal, parent or sibling vis-a-via parent ns
	3 items [153]: self-report, “How many drinks of alcohol have you had in the last 30 days?” (replaced with cigarettes and marijuana). Rate on a 7-point scale to report alcohol consumption (1 = none to 7 = more than 30), cigarette use (1 = none to 7 = more than 20 cigarettes), and marijuana hits (1 = none to 7 = more than 40 hits)	Shin and Hecht, 2012 [94]	Mexican-heritage; middle school students; use Wave 4–6 only	Assessed at wave 4 and wave 6; Did not specify whether sum or other coding for substance use construct	None stated	W4 adult parentification ns, W4 problem-solving parentification ns
**Risky sex**	Two items, self-report, indicating both lifetime sexual activity (yes/no) and sexual intention (5 point scale from 1-disagree strongly to 5-agree strongly)	Sang et al., 2014 [20]	African American and Hispanic mother; HIV-negative daughter; low-income inner-city, recruited in agencies that provided services to HIV-infected women; victims of intimate partner violence, and those in substance use recovery	Daughter report; analyzed separately as two outcomes	N/A	Overall—(intention), Overall ns (lifetime)
	One item, self-report, the number of times they had sex without a condom in the past 6 months	Lester et al., 2010 [72]	Adolescent with HIV/AIDS-infected mothers, or adolescent of neighborhood control mothers	Due to skewed and kurtoses of scores, they were transformed to square root for the analysis	N/A	Role reversal ns
	Two items: (1) Total partners, the total number of different people with whom they had sex in the last 3 months. (2) Sex last 3 months, whether they had sex within the last 3 months (no = 1/yes = 2)	Stein et al., 1999 [95]	Non-infected adolescents of parents with AIDS	Formed latent variable with the two indicators	N?A	Parental role +, Spousal role ns, Nonspecific adult role ns
**Psychological/psychiatric Symptom**	The Trauma Symptoms Checklist (TSC-33) [154]: 33-item, self-report, rate on a 4-point scale ranging from never to very often for psychological symptoms in the last 2 months	Mayseless et al., 2004 [25]	Community sample	Used total distress score across the 5 subscales to evaluate current functioning	0.88	Role reversal ns
	The Comprehensive Assessment of At-Risk Mental States (CAARMS) [155]: Items are scored on a 3-point scale form “absent/false” to “threshold/true”. Subscales assess 7 domains of the psychosis prodrome, severity and frequency/duration for each subscale rate from 0 to 6. The severity of subclinical positive and negative symptoms was calculated by summing the individual severity subscales within each symptom domain to get the severity of subclinical positive and negative symptoms. Sum the severity subscales within symptom domain	Sheinbaum et al., 2015 [93]	College students	Three dimensional scores: paranoid, schizotypal and schizoid; Two subclinical symptom scores: positive and negative symptoms	None stated	Role reversal all +
	The Schedule for Affective Disorders and Schizophrenia—Child version (K-SADS) [156]: structural clinical interview to assess psychiatric symptomatology in three domains: affective, behavioral, and anxiety disorder	Shaffer and Egeland, 2011 [92]	Mother of low socioeconomic status recruited through a public health clinic for prenatal care	Children at age of 17.5 completed the interview; The final composite score for total symptoms is computed as the averages of the natural log transformed scores in each of the three domains	None stated	Childhood BD ns, Adolescent BD + (control for childhood BD)
**Self-esteem**	The Rosenberg Self-esteem Scale [157]: 10-item, self-report, rate on a 4-point Likert scale from 1 (strongly disagree) to 4 (strongly agree). Sum score	Borchet, Lewandowska-Walter, Połomski, Peplińska, and Hooper, 2020 [45]	Polish sample; majority self-identified college students	Adapted Polish version of the scale	0.90	PB +
		Murrin et al., 2021 [78]	College students; typically developing (TD) who reported having a sibling with Autism Spectrum Disorder (ASD)		0.90	PFP ns, SFP ns
		Oznobishin and Kurman, 2009 [85]	College students (Study 1) and high school students (Study 2); both studies include immigrant or Israeli-born		0.86 for both groups	Child dominance ns, Language brokering ns
		Nuttall, Ballinger, Levendosky, and Borkowski, 2021 [80]	Majority of mother sample were non-White (78.4%) and unmarried (74%)	Six items rated on 7-point Likert scale instead; derived from the Rosenberg Self-esteem Scale but it is a subscale in the Psychological Coping Resources	6, 24 and 36 months: 0.82, 70, 89	EP ns, IP ns, Unfairness -
		Wang et al., 2017 [106]	Two elementary school and three high school students		None stated	Coalition ns, Scapegoating −, Parentification +
**Satisfaction with life**	Satisfaction With Life Scale (SWLS) [158]: 5 items, self-report, rate on 5-point scale from 1 (strongly disagree) to 5 (strongly agree)	Çimşir and Akdoğan, 2021 [55]	College students	Turkish version	0.89	PFP ns
		Hooper et al., 2015 [66]	College students	Used rating on a 7-point Likert-type scale from 7 (strongly agree) to 1 (strongly disagree)	0.89	PFP −, SFP −, PBP +
		Oznobishin and Kurman, 2009 [85]	College students (Study 1) and high school students (Study 2); both studies include immigrant or Israeli-born		0.77 and 0.81 for immigrant and for Israeli-born groups	Child dominance +
**Self-efficacy**	The parenting self-efficacy subscale was an expansion of the Psychological Coping Resources measure [159]: 6 items, self-report, rate on a 7-point scale assess parental evaluative cognitions about their abilities to adequately care for their children	Nuttall, Ballinger, et al., 2021 [80]	Majority of mother sample were non-White (78.4%) and unmarried (74%)		0.75 (36 month visit)	EP ns, IP ns, Unfairness +
	Generalized self-efficacy scale [160]: 8 items, rate on a 6-point scale from 1 (not at all true) to 6 (absolutely true)	Oznobishin and Kurman, 2009 [85]	College students (Study 1) and high school students (Study 2); both studies include immigrant or Israeli-born		0.90 and 0.94 for the immigrant and Israeli-born groups.	Child dominance ns, Language brokering -
		Titzmann, 2012 [98]	Ethnic (185) and 197 native German families	Adolescent self-report	0.86	EP ns, IP+

Notes: PFP = parent-focused parentification; SFP = sibling-focus parentification; PBP = perceived benefits of parentification; IP = instrumental parentification; EP = emotional parentification; BD = boundary dissolution; ns: not significant; +: significantly positively related; −: significantly positively related.

### 3.4. Substantive Themes—Quantitative Studies

Most quantitative studies were cross-sectional and only six [19,70,80,92,95,104] were longitudinal studies. As reviewed above, the parentification construct was measured in various ways, including overall level, functionality (instrumental and emotional), roles (parent-focused, sibling-focused), and perceptions (perceived unfairness and perceived benefit). Thus, the link between parentification and outcomes was often contingent upon the dimensions of the parentification being examined.

**Theme** **1.** *Internalizing Problems (Depressive Symptoms, Broad Spectrum, and Distress) are Linked to Parentification in General and Emotional Parentification in Particular; the Perceived Benefit of Parentification is Protective and Linked to Fewer Internalizing Problems.*

The most studied negative mental health outcome was depressive symptoms (25% of all quantitative studies). Across the 21 studies on depressive symptoms, emergent patterns of findings included: (1) a positive association for **emotional** parentification (five positive findings vs. one null finding) and **parent-focused** parentification (four positive vs. two null); (2) null rather than positive associations for **instrumental parentification** (three null vs. one positive) and **sibling-focused parentification** (five null vs. one positive); (3) when parentification was **an overall construct** or studied as role reversal without differentiating any further dimensions, the findings were mixed in its connection with depressive symptoms (four positive vs. two null vs. two negative); (4) **perceived benefit** of parentification, when studied, was consistently negatively associated with depressive symptoms, suggesting its protective role.

Among the studies on internalizing problems (*n* = 11; measured either by forming a latent variable or summing across anxiety and depressive symptoms) and studies on psychological/emotional distress (*n* = 8), only two studies differentiated instrumental from emotional parentification [17,51], two studies exclusively focused on emotional parentification [86,88], and two other studies focused on role-based parentification [95,100]. More **emotional** parentification was consistently linked with internalizing problems or distress, whereas instrumental parentification consistently showed a null association with internalizing problems or distress. Furthermore, role-based parentification was not associated significantly with internalizing problems or distress.

The remaining 13 of the 19 studies on internalizing problems or distress examined the parentification construct as a whole. Researchers interested in internalizing problems or psychological distress likely investigated broader categories of psychopathology (i.e., a spectrum of problems) instead of focusing on more specific subscales of symptoms (e.g., anxiety alone). This tendency may have also spilled over to their treatment of parentification, where the focus is on the overall construct rather than dimensions. Parentification as an overall construct was positively associated with internalizing problems across studies except for one [19], yet role reversal as an overall construct as studied in two papers was not associated with internalizing problems or distress [72,73]. Regarding temporal order, one longitudinal study found that adolescent but not childhood boundary dissolution predicted internalizing problems in adolescence [92].

**Theme** **2.** *Externalizing Problems (Broad Spectrum, Antisocial Behavior) are Associated with Emotional Parentification, and with Overall Parentification to a Lesser Degree; and Neither Substance Use nor Risky Sex was Linked with Parentification Consistently.*

The next most studied outcome was externalizing problems (*n* = 12), including the broad spectrum of externalizing problems (*n* = 8) and aggression/antisocial behavior (*n* = 4). Only one study examined emotional versus instrumental parentification [70] and only one examined maternal emotional parentification [86], both of which found that more emotional parentification was associated with more externalizing problems. Role-based parentification was studied only in one study and was not linked with antisocial behavior [53]. Parentification as an overall construct was positively linked with externalizing problems in four studies [77,82,95,104], yet an almost equal number of studies (*n* = 3) found null results [76,113,161]. The difference in findings cannot be explained by power/sample size because studies that found a null association had comparable sample sizes as the ones revealing a positive association. Together, it suggests that the positive association between overall parentification and externalizing problems was weak at best.

Most of the 11 studies involving substance use as an outcome revealed null findings. Across the 18 associations tested in these 11 studies, only five associations were statistically significant and three were in the unexpected negative direction where more parentification was associated with *fewer* substance use problems [19,72,96]. None of these studies examined instrumental or emotional parentification as they either focused on overall or role-based parentification.

Like findings in substance use, a null finding between parentification and risky sex was more common. Among the six associations examined in three studies on risky sex, the only two significant associations were in opposite directions where more parentification was associated with lower sexual intention [20], and the more parental role was linked with more concurrent risky sex measured as a latent variable [95].

**Theme** **3.** *Despite Many Null Findings, when a Direction Was Reported, It Trended toward Parentification Being Linked with Selected Positive Outcomes.*

Compared to the negative mental health outcomes, very limited studies focused on positive outcomes, and the largest limitation is the small number of studies focused on the same construct to allow a clear conclusion to be drawn. This was true even for the most studied positive outcomes: self-esteem (*n* = 5), self-efficacy (*n* = 3), and satisfaction with life (*n* = 3). Parentification was *not* associated with self-esteem in general as only three of the 11 associations tested in the five studies were significant: Self-esteem was positively associated with perceived benefit [45] and parentification [106], and negatively linked with perceived role unfairness [80]. No consistent findings emerged for efficacy or satisfaction with life across six studies.

Other positive life outcomes such as posttraumatic growth [17,66], happiness [97], school achievement [8], social competence [102], prosocial behavior [42], and empathy [103] were studied no more than twice, but findings tentatively suggested that parentification may be associated with beneficial outcomes. For example, more role-fulfilment from parentification was linked with a higher daily happiness rating [97], more instrumental parentification with better school achievement [8], more parental role with more social competence [102], more parentification with better cognitive empathy [103], and better coping skills 6 years later [19]. However, studies also suggested a lack of association between parentification and happiness [97], emotional parentification and school achievement [8], and parentification and affective cognitive empathy [103]. Future studies on the same constructs are needed to allow any consistent results patterns to emerge.

**Theme** **4.** *Mechanisms and Parentification as Mediators: Three Mediators (Differentiation of Self, Rejection Sensitivity, and Attachment Styles) Emerged for the Effect of Parentification on Various Outcomes; Parentification Mediates the Effects of Various Family Risk Factors on Negative Outcomes.*

Seventeen studies examined mechanisms through which parentification affected the outcomes. The only mediators that were tested more than once were the differentiation of self (defined as “capacity for emotional self-regulation and the ability to regulate the relational impulses of separateness and togetherness” [67,68]), rejection sensitivity [62,63], and attachment styles [41,75,93]. All three constructs were significant mediators connecting parentification with various outcomes. Differentiation of self mediated the effect of perceived unfairness on general mental health outcome [68] and alcohol use [67]. Rejection sensitivity mediated the boundary dissolution effect on false-self behavior [62], and the effect of parentification on same-sex friend intimacy [63]. Lastly, the emotional parentification effect on constructive communication was mediated via anxiety but *not* avoidance adult attachment [75]; paternal parentification effect on adult relationship satisfaction and insecurity was mediated via avoidance/anxiety attachment [41]; and enmeshed attachment mediated the relationship between role reversal and paranoid and schizotypal personality disorder traits [93]. Interestingly, the studies examining the above mediators are largely the work of one research group, except for attachment. The remaining eight studies examined other mediators and outcomes, including quality of life, perceived stress, and communication about alcohol; given the once-off nature of the studies, it is difficult to ascertain patterns.

Seven studies examined parentification as a mediator for other associations, typically associations between other family environments and health/behavior outcomes [39,48,58,59,60,64,88]. Four studies found parentification as a significant mediator connecting family risks with negative outcomes [39,48,59,88], even though the exact family risk factors ranged from domestic violence to parental alcohol misuse, and the outcome also ranged from internalizing problems to aging.

**Theme** **5.** *Exploration of Moderators to Explain Heterogenous Effects: Exacerbating Effect of Maternal Depression and Protective roles of Disclosure of Worries, Social Support, and Religious Service Attendance.*

Heterogeneity of parentification effects on outcomes is organized into two primary categories based on features of moderators: demographic features, mostly sex, and race, versus non-demographic characteristics (e.g., social support). Nine studies examined non-demographic moderators for the parentification effect, and exacerbating and buffering moderators were identified. Maternal depression appeared to exacerbate the negative effect of parentification on adjustment [89,111]. Some protective factors emerged that either buffer the negative effect of parentification or facilitate the effect of parentification on positive outcomes, including disclosure of worries, social support, and religious service attendance. For example, more sibling- and parent-focused parentification was associated with negative interaction with siblings and less wellbeing, but only at low *disclosure of worries* about autistic sibling to parents [78]; language brokering was linked to less self-efficacy, but only at low *parental/social support* [85]; parentification was linked with distress, but only at low levels of *social support* [101]; more perceived unfairness was linked to more alcohol use for those not attending *religious service* [67].

In summary, parentification, particularly emotional parentification was linked with more depressive symptoms and internalizing problems. Findings on externalizing problems were weaker and revealed null associations more often than not for substance use and risky sex. Positive outcomes of parentification were much less investigated, but research suggested a potential positive link between parentification and desirable life outcomes (e.g., coping skills). Differentiation of self, rejection sensitivity, and attachment styles were promising mechanisms that elucidate the link between parentification and negative health outcomes. Parentification also mediated the associations between various family risk factors and adverse outcomes. Lastly, while exploring the heterogenous effect of parentification, researchers explored not only exacerbating factors (e.g., maternal depression) for the negative impact of parentification, but also buffering factors (e.g., social support) that exerted more of a protecting role to buffer the negative impact of parentification and/or to facilitate the positive outcomes of parentification.

### 3.5. Substantive Themes—Qualitative and Mixed Methods Studies

Across the body of 14 qualitative and mixed methods studies, parentification was not consistently related to negative or positive outcomes. Ten studies reported both positive and negative outcomes, two studies reported only positive outcomes [28,36], and two studies reported only negative outcomes [31,35]. Five themes emerged across the studies.

**Theme** **1.** *Personal Growth and Strengthened Sibling Relationships Come from Adversity*.

Reported positive outcomes from parentification included personal growth (e.g., emotional intelligence, prioritizing others, social skills, independence) and strengthened sibling relationships. In seven qualitative and mixed methods studies, participants reported personal growth as a positive outcome of their parentified experiences. For some, personal growth meant enhanced *emotional intelligence*, characterized by increased perspective-taking, enhanced empathy, and a desire to understand and care for others [28,29,34]. Personal growth was also defined as *prioritizing others* above self-interests and the development of social skills [26]. For others, personal growth was described as developing *greater independence* [32], *agency* [26], or *grit* [33].

A strengthened relationship between and among siblings was a means of relational coping, and was particularly important in contexts where youth were physically or socially isolated (especially through stigma), experienced mothers’ mental health concerns, or were abused, neglected, or witnessed domestic violence [26,27,34]. Strengthened relationships with siblings were both an outcome of parentification and helped reinforce and promote personal growth. When building stronger sibling bonds, these youth developed empathy, understanding, acceptance for others, and other social skills (e.g., positive, emotional connections, providing social support); these all contributed to them aspiring to have future careers in human services fields to care for others and striving to break down mental illness stigma [26,34].

**Theme** **2.** *Self-Preservation Mechanisms—Self-sacrifice, Distancing, and Balancing Desire for Closeness*.

To manage their experiences, parentified children developed numerous coping strategies, many of which were suboptimal by conventional standards but adaptive for their situation [25,30,32,35,37]. Some youth expressed a self-imposed heightened sense of protecting parents from worry and stress [32]. For example, parentified youth did not speak about abuse by employers because of worry about losing employment and no longer being the family breadwinner [35]. This was a form of emotional self-sacrifice to promote instrumental resources for the family, and it also demonstrates how self-sacrifice in one domain (downplaying emotional needs or workplace silence) serves as self-preservation in another domain (minimizing the effect of additional stress on parents or potentially losing household income).

While some youth protected their parents by not sharing worrisome and stressful matters, some youth chose to emotionally distance themselves from mothers to avoid criticism and retraumitization [31,34,37] Mother-child relationship quality influenced how much and why youth shared information: protect mothers (self-sacrifice) or protect themselves (distancing). The participants who preferred more distance from mothers limited contact to avoid conflict despite the tension of wanting closeness.

Some youth found ways of balancing family demands with self-care and individual needs. For instance, some scheduled time to be alone or interact with friends. Chee and colleagues [28] described this as “children’s parentification was found to be a process involving intense yet subtle dynamics of cooperation, negotiation, and resistance” (p. 209).

**Theme** **3.** *Premature Transitions, Compromised Human and Social Capital, Lost Childhood, and Difficulty Forming Adult Relationships*.

Some parentified youth discussed how greater maturity and independence relative to peers prepared them for a transition to adulthood, but these positive attributions were often accompanied by negative attitudes about forfeited childhoods and compromised adult outcomes. Participants described a “lost childhood” marked by insufficient time to engage in play and other activities with same-aged mates [32], and basic attachment-related needs for comfort were not met in childhood [25,31].

Due to premature transitions into adulthood roles, parentified youth commonly experience compromised human capital in the form of incomplete educational attainment due to school dropout or low attendance, limited to no time with peers to develop relationships and friendships, and early transition into parenthood partially the result of early or risky sexual behavior and substance use [29,32,38]. Compromised educational attendance and attainment was a common outcome for parentified youth [27,29,32,33,35]. Forming relationships with others is sometimes difficult due to limited time, issues of trust and fear, for example, parentified girls avoided emotionally intimate relationships and did not accept support from others because they fear being parentified by support persons as a quid pro quo, meaning they will owe that person for any support received [31].

**Theme** **4.** *Suboptimal Mental and Physical Health among Parentified Youth*.

Whereas some youth perceive and respond to parentified experiences with positive perceptions and coping strategies, other parentified youth experience anger, loneliness, resentment, feelings of being overwhelmed, and substance use [32,33,38]. Either by parent reports or youth self-reports, youth experienced suboptimal mental and physical health outcomes. Mental and physical health concerns include depression, anxiety, worry, toxic stress and trauma, dementia, domestic violence, substance abuse, displacement (war, foster care, orphaned), poverty, and HIV [25,31,34].

The suboptimal physical health outcomes of parentification could be exacerbated by co-occurring risk factors that many parentified youth experience. These co-occurring risk factors include a lack of access to affordable health care, healthy and stable housing, sufficient and quality water and food; harsh or abusive working conditions; domestic violence, and harsh parenting [25,27,35]. Addressing these needed resources and supports may prevent or mitigate some of the negative outcomes of parentification.

**Theme** **5.** *Modifying Influences: Perception, Acknowledgement, Initial Competencies, and Supports Make the Difference between Floundering, Resilience, and Thriving*.

The role of perceived fairness [36], being rewarded with statements of appreciation [28,36], positive relationships with parents [34], and receiving community, social, and service supports [27,34] influenced whether youth experienced floundering, surviving, resilience, or thriving outcomes. Youths’ perceptions about their experience of parentification (e.g., fair, positive, unfair, loss) and the degree to which emotional and resource supports are provided (e.g., appreciation, food, housing) influence the trajectories they traverse—resilience and thriving or surviving and succumbing [36,86]. A lack of parental emotional support in childhood may take the form of parents dismissing or underplaying the children’s parentification experience, and providing less warmth and support [86], assuming children should and will take on parenting responsibilities, which could dismantle children’s trust in parents and other adults to care about them.

In contrast, small displays of appreciation for children’s contributions to the family were related to positive outcomes for children (e.g., confidence) [28,36]. Among orphaned youth, some youth were reported to be resilient by going to night school to earn their degree and providing food and housing for siblings with community assistance; and these resilient youth had more social capital and support from community members, siblings, and peers [27]. Further, support from family and friends alleviated the negative impact of parentification, as did grit (personal strength) and sense-making or meaning-making, which depended on cultural context [33]. Initial competence and locus of control levels also help determine if children experience resilience [36].

### 3.6. Integrated Themes—Quantitative, Mixed, and Qualitative Studies

Theme alignment across qualitative, quantitative, and mixed methods studies is presented using an integration matrix (Table 6). This section focuses on four overlapping themes. Two quantitative themes map onto one qualitative theme, resulting in a total of four integrated findings.

First, across qualitative, quantitative, and mixed methods studies, positive outcomes in the form of resilience and positive coping were reported. Influential factors include the type of parentification—instrumental and the strengthening of relationships such as those with siblings. Although specific outcome constructs varied, these factors confer feelings of contributing to the household or family and opportunities to develop skills related to empathy, agency, esteem, and prioritization of others.

Second, youth employed several strategies to protect themselves or their parents from additional trauma. Mechanisms include not sharing or disclosing information with parents that would increase their worry and stress; distancing themselves from parents, even though they experience a tension between desired closeness and avoiding additional suffering. The family environment (e.g., maternal mental health or support) served to either protect or place youth at a greater risk for negative outcomes.

Third, parentified children experienced negative outcomes, including internalizing problems (e.g., depression, anxiety), externalizing problems (e.g., substance use, sexual risk-taking), and physical health (e.g., physical abuse, poor nutrition). Relationships between parentification and physical health and externalizing outcomes were more present in qualitative accounts than quantitative statistical tests.

Last, reports of positive or negative outcomes were influenced by afflicted youths’ characteristics (e.g., self-differentiation, rejection sensitivity), attachment style, and perceptions of the benefits or fairness of adult responsibilities. These factors moderate how parentification relates to outcomes—protective/buffering or increasing risk. The buffering effect of other social supports (e.g., community members, teachers, and others) was reported to reduce the outcome severity youth experienced. Perceived benefits and fairness were associated with fewer negative mental health outcomes. These factors influenced the type of trajectory youth traversed.

Although not substantial enough to qualify as themes for qualitative or quantitative studies due to a dearth of longitudinal studies of multiple generations, there was mention of intergenerational impacts of parentification in several studies. For example, the mother’s role reversal with her mother predicted the mother-toddler role reversal over and above attachment, suggesting a need for preventive interventions to address not only attachment disorganization, but also the mother’s own history of parentification [74]. In another study, among a high-risk community sample of first-time mothers, maternal history of destructive parentification had an indirect effect on child externalizing behavior through warmth and responsiveness [82]. In yet another study, Tedgård and colleagues studied how parents’ substance abuse translated into instrumental and emotional parentification of their children and the intergenerational impact on parenting. These parentified youth had an attribution bias toward danger and threat that resulted in being overprotective and overbearing of their own children [38].

**Table 6:** Integration Matrix of Themes by Study Design.

	Qualitative/Mixed Themes	Quantitative Themes	Alignment/Integration
*Resilience, Positive Outcomes, and Self-Preservation*	*Personal Growth and Strengthened Sibling Relationships Come from Adversity* Emotional intelligence Prioritizing others Independence, agency, grit	*Positive Outcomes of Self-Esteem, Efficacy, and Satisfaction with Life had many null findings; when a direction was reported, it trended toward parentification being linked with some positive outcomes. (Quant theme 3)* Instrumental parentification and perceived benefit linked to more positive outcomes Too few studies examined the same positive construct for specific patterns, but across constructs, resilience emerged.	Across qualitative, quantitative, and mixed methods studies, positive outcomes in the form of resilience and positive coping were reported. Influential factors include the type of parentification—instrumental and the strengthening of relationships such as those with siblings. Although specific outcome constructs varied, these factors confer feelings of contributing to the household or family and opportunities to develop skills related to empathy, agency, esteem, and prioritization of others.
	*Self-Preservation Protective Mechanisms—Self-sacrifice and Distancing* Youth utilized several strategies to protect themselves or their parents from additional trauma.	*Exploration of moderators to explain effect heterogeneity: exacerbating effect of maternal depression and protective roles of disclosure of worries, social support, and religious service attendance (Quant Theme 5).* Disclosure of worries, social support, and religious service attendance either buffered the negative outcomes of parentification or facilitated the positive outcomes. Maternal depression exacerbated the negative outcomes of parentification.	Youth employed several strategies to protect themselves or their parents from additional trauma. Mechanisms include not sharing information with parents that would increase their worry and stress; distancing themselves from parents even though they experience a tension between desired closeness and avoiding additional suffering. The family environment (e.g., maternal mental health or support) served to either protect or place youth at greater risk for negative outcomes.
*Compromised Development or Negative Outcomes*	*Premature Transitions, Compromised Human and Social Capital, Lost Childhood, and Difficulty Forming Adult Relationships*	Not measured or mentioned.	Not applicable.
	*Suboptimal Mental and Physical Health among Parentified Youth—Vulnerabilities and Stacking Risk Factors* Anger, Loneliness, Resentment, Feeling Overwhelmed, Poor Nutrition, Lack of Stable Housing, Physical Abuse, and Substance Use Responses	*1. Internalizing Problems Linked to Parentification in General and Emotional Parentification in Particular (Quant Theme 1)* *2. Emotional Parentification Related to Broad-Spectrum Externalizing Problems (Quant Theme 2)*	Parentified children experienced negative outcomes in the areas of mental health or internalizing problems (e.g., depression, anxiety), externalizing problems (e.g., substance use, sexual risk-taking), and physical health (e.g., physical abuse, poor nutrition). Relationships with physical health and externalizing outcomes were more present in qualitative accounts than in quantitative statistical tests.
*Moderators and Measurement Implications*	*Modifying Influences: Perceptions, Acknowledgement, Initial Competencies, and Supports Make the Difference between Floundering, Resilience, and Thriving* Perceived fairness, emotional and resource support, and acknowledgment of youths’ assistance can impact trajectory directionality.	*Mechanisms and Parentification as Mediators. (Quant Theme 4)* Quantitative studies suggest differentiation of self, rejection sensitivity, and attachment style serve as mediators. Parentification mediates the effects of various family environmental risk factors on negative outcomes Perceived unfairness and benefits impact whether positive or negative outcomes are experienced.	The report of positive or negative outcomes was influenced by afflicted youths’ characteristics (e.g., self-differentiation, rejection sensitivity), attachment style, and perceptions of the benefits or fairness of these adult responsibilities. These factors mediate how family risk factors relate to outcomes—protective/buffering or increasing risk. The buffering effect of other social supports (e.g., community members, teachers, and others) was reported to reduce the outcome severity youth experienced. Perceived benefits and fairness were associated with fewer negative mental health outcomes. These factors influenced the type of trajectory youth traversed (e.g., severity degree and positive/negative nature).

## 4. Discussion

### 4.1. Summary of Findings

A majority of studies relied on adult retrospective accounts, participants tended to be mostly female or college students, and there was representation from six continents, demonstrating this is a global phenomenon. This systematic review revealed that parentification may have both positive and negative impacts on coping, family relationships, mental and physical health, and human and social capital. Altogether, this work implies that parentified youth need support, especially those experiencing early-onset parentification when their cognitive and emotional systems are still immature. The early intervention of social, emotional, and cognitive support could mitigate adverse impacts stemming from sources of parentification, such as parent mental and physical health challenges, exposure to domestic violence, war, and unstable housing.

A few overlapping themes emerged from quantitative and qualitative studies. These include positive outcomes of enhanced coping and resilience; negative outcomes of suboptimal mental health and problem behaviors; protective effects of perceived benefits and social support; and mediating roles of self-preservation mechanisms. The results indicate that parentification is a complex process in which linear explanations may not be sufficient and different mediators and moderators should be considered.

We have found parentified children experience various suboptimal outcomes in adulthood [15,16,17]. One reason is that these children perceived their obligatory adult roles negatively, as unfair and “robbing” them of their childhood, and experienced stress, role overload, and resentment. Another explanation is that parentified youth and their siblings are expected to prematurely assume adult-like responsibilities during developmental stages (e.g., childhood, adolescence) marked by immature brain development, which is not fully optimized until age 25 [162,163]. In addition, brain development is negatively impacted by early and chronic stressors [164], such as parentification. These youth may have limited opportunities to learn, observe, and utilize positive coping strategies typically derived from parental influences. Rather, parentified youth are disadvantaged by a lack of emotional and instrumental support from parents who cannot meet their emotional and psychological needs [25,31].

Resilience among parentified youth is not as well documented, in part due to quantitative study focus bias on negative outcomes. Qualitative studies in this review, however, provide insight into how resilience develops from parentification experiences. The studies suggest that it may be important to recognize that some parentified youth may emerge into adulthood relatively unscathed or robust against adversities they experienced. Unfortunately, this area of the parentification literature is scarce, and we currently know little about the positive outcomes of parentification and the mechanisms underlying resilience.

### 4.2. The Role of Culture and Context

Across the 95 studies, 42% represented non-US countries: Nine of the 14 (64%) qualitative or mixed studies and 31 of the 81 (38%) quantitative studies. The sources, meanings, and practices of parentification may vary across these contexts. For example, countries with higher prevalence of infectious diseases such as HIV/AIDS, that increase the likelihood of parental death, may result in fewer adult supports for children who are orphaned or lose one parent. Countries experiencing pressures for flight may result in refugee families whereby children are expected to serve as language brokers and workers because it is easier for them to learn the language of new countries. These language brokers may experience *both* heightened instrumental *and* emotional parentification that may be further exacerbated depending on the child’s age. For example, if youth are particularly young (pre-pubescent; a time when learning multiple languages is easier), they may be asked to translate adult conversations that require an emotional maturity they do not have, making these conversations particularly difficult for youth.

The fact that the parentification inventory has been translated into many languages and has demonstrated reliability and validity in studies across continents—North America, Australia, South America [165], Africa [166], Europe [167], and Asia [55], suggests the phenomenon of parentification is global. Policies and resources provided to parentified children also vary by countries [168]. Although some studies reviewed here did discuss and interpret their findings in light of the cultures where they were embedded, the cross-culture comparison was not explicitly tested because studies were generally carried out in one country and precluded such empirical tests of cultural differences. For example, these studies had discussed the readiness of youth taking on care-giving roles in a more collectivistic culture in Korea [54], the implications of relatively higher degree of emotional interdependence in Turkish culture that affects parentification [55], and the impacts of historically collective and communal focus versus the modern individualistic orientation in Jewish Israel culture on parentification and enmeshment [63]. In short, none of the studies in this review explicitly conducted cross-national comparisons of parentification experiences and associations with outcomes, a gap future studies should address.

### 4.3. Dimensions of Parentification and Promising Research Directions

One challenge involved in integrating findings across quantitative studies concerns the dimensions of parentification being investigated. Many studies used standardized measures either just on the *functions* parentification served (emotional versus instrumental) or just on *roles* (sibling-, parent-, or spouse-focused). Results were not directly comparable across studies focused on function versus roles of parentification.

Emergent quantitative findings suggested emotional parentification may be more detrimental than instrumental parentification, in that emotional parentification was consistently linked with depressive symptoms and a broader spectrum of internalizing and externalizing problems. In contrast, no clear pattern for the link between role-based parentification measures and outcomes emerged. Emotional parentification may be more overwhelming for youth, who at the age of parentification onset, were not developmentally equipped with the necessary skills to adequately cope with the imposed emotional demand. As seen in qualitative studies, parentified children resort to suboptimal coping strategies to protect themselves and their parents from additional emotional trauma (e.g., not sharing about stressors, self-sacrifice, and distancing).

Instrumental parentification, on the other hand, may be more predictable and prescribed (e.g., working hours, household responsibilities), making it more likely to be within the youth’s capabilities, even if it is demanding in time and effort. From an evolutionary psychology perspective, youth taking on instrumental responsibility (e.g., caring for younger siblings, doing house chords) to contribute to family economy reflects our prehistoric past as a species, since children and adolescents took on varies duties in tribal societies that make up most of the human cultures. Being able to make contributions to the family in an instrumental way may be psychologically favorable for self-esteem development; hence, explaining why instrumental parentification has not been found consistently linked to a host of negative outcomes. The mixed findings for role-based parentification may be explained because any of them (e.g., sibling- or parent-focused) could be any combination of emotional or instrumental parentification elements. If the association with mental health indeed resides in emotional parentification and it was not directly measured by a role-based instrument, then it is not surprising to see mixed findings for role-based parentification. Given that emotional parentification was more consistently associated with suboptimal mental health, focusing on emotional versus instrumental parentification may present new research avenues for identifying mechanisms, such as focusing on emotion regulation, positive coping, and social support as mediators for emotional parentification [27,32,34]. In contrast, role-based parentification likely limits further formulation of mechanisms, as it may be difficult to draw on existing psychological theories to hypothesize why and how parent-focused and sibling-focused parentification have differential outcomes, and how they affect different mediators to exert their impact on mental health outcomes. We argue that the studies operationalizing parentification through emotional versus instrumental dimensions will advance the study of parentification in a more fruitful direction.

### 4.4. Perceptions and Perceived Benefits of Parentification

The significance of perceptions emerged from both quantitative and qualitative studies. As revealed in the results, perceived benefits of parentification were generally linked with more positive outcomes and less negative outcomes across studies. We want to highlight an important conceptual issue that has not received enough consideration: should perceived benefits be treated as a dimension of parentification equally as function-based parentification or role-based parentification, or should it be conceptualized as a moderator or mediator of the impact of parentification on outcomes? Currently, only the former conceptualization has been explored, largely because the perceived benefits of parentification have been measured as a subscale in parentification measures. Researchers typically conduct main effect analyses to examine how perceived benefits were linked to outcomes, and no existing quantitative research examined it as a moderator for the effects of parentification. Perceived benefits of parentification being treated as a subscale of parentification may have hindered efforts to explore how perceptions are a moderator or mediator for the parentification effects.

The reverse causation issue was not addressed and rarely discussed in research that found positive outcomes of perceived benefits. Positive outcomes may *cause* higher ratings of perceived benefits of parentification in quantitative research and more positive perceptions of parentification in qualitative studies. This issue is particularly salient in retrospective research where individuals may interpret their past parentification experiences differently. We firmly believe it is critical to understand individuals’ perception of benefits from their experiences, yet this should be separated from measuring the parentification experience itself. By clearly conceptualizing perceived benefits as separate from parentification and identifying ways of dealing with the potential reverse causation issue, we can better advance our understanding of parentification and its heterogenous impacts.

### 4.5. Adverse and Positive Life Outcomes

Although over three-quarters of the quantitative studies focused on the negative outcomes of parentification, surprisingly few consistent findings could be identified. Except for depressive symptoms, broad spectrum internalizing and externalizing problems, and substance use, most constructs were studied only a handful of times and some constructs were almost exclusively studied by the same research groups. To advance an area of knowledge, individual studies should build on the existing literature and situate themselves well into the larger literature. Yet, many “one-off” studies were observed, the findings of which were neither replicated nor carried forward. Future research is needed to address these problems and meaningfully build the parentification literature with more depth and consistent use of outcome measures and constructs rather than casting a wider net by adding more “one-off” outcomes. For example, there were consistent findings for emotional parentification and depressive symptoms. Future research can build upon these replicated findings by identifying various pathways of *how* emotional parentification exerts its effect through physiological or psychological mediators (e.g., stress cortisol reactivity, resting heart rate variability for emotion regulation capacity, coping strategies). Focusing on the *how* and *why* will move the field further forward and provide insights into supports, programs, and interventions needed to mitigate the negative outcomes that these parentified youth experienced.

It is clear from this review that resilience remains understudied. Less than 15% of the quantitative studies included positive or resilient outcomes. Although most qualitative studies were open-ended, none explicitly posed research questions addressing the positive outcomes of parentification. Nonetheless, studies presented promising findings that parentification or dimensions of parentification were linked with beneficial outcomes such as coping skills, social competence, or general well-being. The “one-off” nature of parentification research is even more pronounced when looking at studies that measured positive outcomes. Parentification research can benefit from the positive youth development and positive psychology approaches by including resilience and positive coping into their research designs.

### 4.6. Adopting a Developmental and Systems Perspective

Only a handful of studies examined parentification from the entire family and at multiple time points. Fathers and sons are undoubtably understudied within the parentification literature. From a systems perspective, examining dyads, triads, or the family unit is important given the ecological and biosocial implications of parentification within a family system. Further, from a behavioral genetics perspective [169], children raised in the same household have shared and non-shared experiences and biological vulnerabilities (genetic predispositions) that are important considerations in understanding the consequences of parentification. The source of parentification may be parents’ mental health conditions, such as depression, schizophrenia, and narcissism [31,34], implicating biological and social vulnerabilities for children to experience similar or related conditions. With the increased prevalence of restructured families where stepsiblings, half-siblings, and adopted siblings are more commonplace, the context and implications of parentification may vary. Future studies could explore parentification as a function of different levels of sibling relatedness, which contributes to differentiating gene (biological) and environmental (behavioral) influences, helping to clarify shared genetic vulnerability and environmental influences.

From a socialization perspective, studying parent-child dyads may reveal how parentification impacts and is impacted by relationship quality. As noted above, one theme that emerged from qualitative studies was how parentified children withheld information from parents for reasons of protecting parents from additional worry and self-preservation through maintaining independence and emotional distancing. Further, parentified youth are disadvantaged by a lack of emotional and instrumental supports from parents who cannot meet their emotional and psychological needs [25,31]. The role of parent-child relationship quality in predicting, being predicted by, and moderating parentification outcomes was not directly or explicitly measured in the included studies, and is an area for future research, especially longitudinal designs.

In addition, prospective studies are important given the developmental implications of parentification. Qualitative study findings highlight compromised educational attainment as a negative outcome of parentification, yet most quantitative studies that we reviewed were conducted with college-going samples and cross-sectional, which together raise further concern that youth who did not go to college are underrepresented. Longitudinal studies would capture important characteristics and contexts of parentification, including onset timing, duration of experience, short-term vs. long-term adaptations, sleeper effects, and retrospective reframing. These factors should be considered to better understand how the effects of parentification may vary based on developmental periods.

Adopting a developmental and systems perspective to research parentification requires a large sample size, longitudinal design, and a focus beyond college students, which is lacking in the current research. For example, the majority of quantitative studies had relatively small sample sizes (less than 300 participants), suggesting statistical power may be a concern already, even with a cross-sectional design, for the number of dimensions studied, multiple tests run, and sophisticated analyses including latent variable, mediation, and moderation modeling.

### 4.7. Youth Measure and Non-Linear Modeling and Explanation Considerations

From a measurement perspective, measures specific to youth reports warrant additional refinement, including improved reliability. These measures have been adapted from adult versions and few studies have utilized them. It may be useful to use cognitive interviewing and mixed methods approaches to develop these youth measures to ensure their reliability.

Related to modeling, considering non-linear explanations and descriptions of parentification is recommended. A linear explanation of the relationship between parentification and outcomes does not capture the nuances of the phenomenon. Parentified individuals report both positive and negative perceptions and outcomes. For the majority of qualitative studies (*n* = 10), both positive and negative outcomes were found (e.g., parentified children reported both personal growth and high school dropout [29]). Callaghan and colleagues refer to this as an “empowering and constraining” duality (p. 662) [26]. Further, as Chee and colleagues highlight [28], curvilinear relationships between functioning and parentification need to be explored, whereby those not at extremes adapt well. A different approach warranting future exploration in quantitative research is to consider the combined effects of various parentification domains. For instance, youth reporting high emotional coupled with high instrumental or low emotional coupled with high instrumental may report more positive outcomes compared to youth experiencing high emotional—low instrumental parentification. There may be a buffering effect of instrumental parentification (associated with leadership skills, esteem from responsibility fulfillment, and achievement) not otherwise captured if we modeled dimensions of parentification separately. The quantitative studies reviewed rely heavily on linear modeling and assumptions, which may need to be relaxed to ensure a more holistic understanding of the parentification phenomenon.

### 4.8. Strengths and Limitations

Our review has several limitations. First, only one mixed-method study was identified with our inclusion criteria. This necessitated its grouping with qualitative studies and limited our ability to contrast this study design with quantitative and qualitative studies. Second, although 81 quantitative studies were identified and included in the review, about 20% of these studies contributed less weight to the synthesized findings because of the heterogeneity in outcome constructs, where outcomes were only investigated once or twice. Third, conclusions rest largely on a majority of studies that used cross-sectional designs and employed non-probability-based sampling. This precluded causal inference and may introduce selection effects. Nonetheless, we identified several meaningful gaps and important directions for future studies.

These limitations should be balanced with the strengths and significance of our review. It is the first systematic literature review to examine parentification studies with outcomes. This study was inclusive of qualitative, mixed, and quantitative studies from across the world and utilized three databases to ensure comprehensiveness. MMAT provided additional analysis of study bias and quality, evidencing that the risk of bias is minimal. The use of mixed methods integration analyses enabled the comparison of themes across different study designs. Five qualitative and four quantitative themes overlap, suggesting consistency and congruity in findings despite outcome measures varying. Policy and programmatic recommendations were gained.

## 5. Conclusions

The study of parentification has public health significance and practical implications. Although parentification may confer positive outcomes, its relationship with resilience has been grossly understudied. The predominantly negative consequences of parentification are far-reaching, including the parentified individual, the immediate family, and intergenerationally, speaking to the need to further understand its causes, consequences (both positive and negative), typologies, and intervention. Many parentified youth experience compromised human, social, and financial capital that strongly predict long-term outcomes for these youth and their families, including under- and un-employment and mental and physical health disparities. Identifying and providing needed support early and often is needed.

In the US, demographic changes related to family structure (e.g., increasingly unstable, complex, and “fragile” families), economic demands, and health disparities have and will further increase the number of parentified youth. Factors contributing to this role strain include household poverty, siblings with additional needs, parental illness, parental divorce or separation, and other stressors affecting youth and families. In the US, COVID-19 has disproportionately negatively impacted marginalized racial and socioeconomic strata with Black adults and youth more likely to experience compromised household income, housing stability, and mental and physical health, [170,171] which are sources of parentification pressures for children. These pressures are not isolated to the US, but rather are globally relevant with additional concerns in other countries including wars, humanitarian atrocities, and epidemics that have far-reaching impacts and disproportionately impact the most vulnerable and lowest-resourced families. These circumstances create a contagion of risk factors with youth particularly vulnerable to parentification, whereby children assume adult responsibilities. This study elevates the importance of this topic and provides feasible and actionable substantive and methodological recommendations for future studies. Further, this review highlights (a) the need for social support for these youth, and (b) investment in programs and policies that invest in youths’ ability to increase their human (e.g., education) and social (e.g., peer relationships) capital and physical health (e.g., medical, nutrition) while helping to provide for their households (e.g., rent, food).

The implications of parentification expand beyond the parentified individual (e.g., psychological, cognitive, and physical health outcomes) to the family of origin (e.g., sibling outcomes) and future generations through an intergenerational transmission [1]. The fact that the prevalence of parentification in the US is unknown, yet is greater than 30% of youth in Poland and impacts family members—horizontally to siblings and vertically to offspring—further amplifies the public health need for studying this phenomenon, negative sequelae, and opportunities to promote resilience for youth and families.

## Figures and Tables

**Table 1:** Search Algorithms and Articles Generated by Database.

Search Terms (Same in All Three Data Bases)	Articles Count	Database
(spousif* or parentif* or adultif* or “role reversal”) AND (outcome* or resilien* or thriv* or stress or react* or benefit* or impact* or effect* or affect* or positiv* or negativ*)	227	PsycInfo
	313	Academic Search Complete
	571	Web of Science

**Table 2:** Data Item List, Definition, and Format.

Data Item	Definition
Study Citation Information	Full citation; separate columns for year and title
Population	Description of the target population (e.g., adults, young adults, children)
Location	Country context
Participants	Description of sample characteristics and sampling
Methods	Description of data collection procedures
Focus of the Study	Specify outcomes of focus (e.g., mental health, externalizing problems)
Type of Research	Specify study design including three categories (qualitative, quantitative, mixed methods) and other relevant details
Parentification Measures	Specify parentification measures used (name and scale/subscale description)
Parentification Constructs	Specify parentification constructs measured including instrumental, emotional, perceptions
Mediators, Moderators, Mechanisms	Describe any mediators, moderators, or mechanisms explored (e.g., coping, social support)
Analytic Approach	Description of analyses conducted
Key Findings	Summary of major findings reported by authors
Positive Outcomes	Description of positive or resilient outcomes as well as significance level and direction
Negative Outcomes	Description of negative or floundering outcomes as well as significance level and direction
Strengths	Summary of study strengths
Limitations	Summary of study limitations
Future Directions	Summary of future directions
Round 1 Notes	Questions or impressions about relevance
Round 1 Site Reviewer	Institution of reviewer
Round 2 Tier	Random split articles into first, second, and third tier for sequence of review
Round 2 Updates	Indicate whether data extraction fields were updated upon verification
Round 2 Notes	Specified data extraction field changes; additional questions about relevance or interpretation of outcomes
Round 3 Site Reviewer	Assessed study quality using MMAT; reviewers independently rated criteria; 10% were cross-validated
Round 3 Notes	Questions or discrepancies in criteria rating; final articles to exclude were noted; misclassification of study design was resolved and appropriate MMAT applied

Note: Data items were in text format except type of research, institution of reviewer (UIUC or GSU), Round 2 tier, Round 2 updates (yes/no), and Round 2 site reviewer.

## Data Availability

No new data were created or analyzed in this study. Data sharing is not applicable to this article.
